# Explaining Evaporation-Triggered Wetting Transition Using Local Force Balance Model and Contact Line-Fraction

**DOI:** 10.1038/s41598-018-37093-6

**Published:** 2019-01-23

**Authors:** Rama Kishore Annavarapu, Sanha Kim, Minghui Wang, A. John Hart, Hossein Sojoudi

**Affiliations:** 1 Department of Mechanical, Industrial, and Manufacturing Engineering (MIME), The University of Toledo, 4006 Nitschke Hall, Toledo, OH 43606 United States; 2 Department of Mechanical Engineering, Massachusetts Institute of Technology (MIT), 77 Massachusetts Avenue, Cambridge, MA 02139 United States; 3 Department of Chemical Engineering, Massachusetts Institute of Technology (MIT), 77 Massachusetts Avenue, Cambridge, MA 02139 United States

## Abstract

Understanding wettability and mechanisms of wetting transition are important for design and engineering of superhydrophobic surfaces. There have been numerous studies on the design and fabrication of superhydrophobic and omniphobic surfaces and on the wetting transition mechanisms triggered by liquid evaporation. However, there is a lack of a universal method to examine wetting transition on rough surfaces. Here, we introduce force zones across the droplet base and use a local force balance model to explain wetting transition on engineered nanoporous microstructures, utilizing a critical force per unit length (FPL) value. For the first time, we provide a universal scale using the concept of the critical FPL value which enables comparison of various superhydrophobic surfaces in terms of preventing wetting transition during liquid evaporation. In addition, we establish the concept of contact line-fraction theoretically and experimentally by relating it to area-fraction, which clarifies various arguments about the validity of the Cassie-Baxter equation. We use the contact line-fraction model to explain the droplet contact angles, liquid evaporation modes, and depinning mechanism during liquid evaporation. Finally, we develop a model relating a droplet curvature to conventional beam deflection, providing a framework for engineering pressure stable superhydrophobic surfaces.

## Introduction

Superhydrophobic surfaces^1^ have attracted increasing research interest due to numerous applications in engineering^2–4^, biomedical fields^5–7^, and daily life^8^. Inspired from nature (for example lotus leaf ^9^) researchers have tuned surface roughness^10–13^, surface chemistry^14^ and sometimes both the characteristics^15^ for fabricating superhydrophobic surfaces. A superhydrophobic surface generally exhibits a large water contact angle (WCA, greater than 150°) and a small contact angle hysteresis (i.e. roll-off angle less than 5°). In general, the contact angle hysteresis (CAH) of rough surfaces depends on the droplet’s state. The two most commonly exhibited droplet states are a Cassie-Baxter (CB) state^16^, where the droplet suspends on top of the microstructures forming a liquid-air-solid composite interface and a Wenzel (W) state^17^, where the droplet impales into the microstructures and fully wets the surface. The latter displaying larger contact angle hysteresis due to the pinning of the three phase contact line (TPCL)^18^. For many applications, like self-cleaning^19^, anti-icing^20–24^, biofouling control^25^, drag reduction^26^, delaying frost growth^27^ and tunable drug delivery^28^, maintaining the stable superhydrophobic Cassie-Baxter state is of primary importance, as these properties are lost when the droplet transits to the Wenzel state. Many studies have focused on stabilizing the wettability of the superhydrophobic surfaces and on the wetting-transition mechanism, through experimentation^29–32^, numerical simulation, and theoretical analysis^33^. There are also a category of superhydrophobic surfaces exhibiting a Cassie-Baxter state with large water contact angles (WCA, greater than 150°) and large contact angle hysteresis (greater than 90°), referred to as sticky superhydrophobic surfaces^34^. The increased adhesion observed at higher area-fractions (f)^35^ on non-sticky (or normal) superhydrophobic surfaces is different from the inherent adhesiveness of the sticky superhydrophobic surfaces. For the first case, the increase in adhesion is due to an increase in the contact line-fraction along the three phase contact line (TPCL), whereas for the latter the higher adhesion is due to the tip geometry^36,37^ and nanopores^38^ on the microstructure. The wetting-transition mechanism and contact-line depinning are strongly affected by the nature of the superhydrophobic surface (whether sticky or non-sticky). In this work, we study the wettability of the non-sticky (or normal) superhydrophobic surfaces. 

Several wetting-transition mechanisms^39^ are proposed based on the energy barrier^29,32,40^ or the critical Laplace pressure^30,41^. Despite many efforts, there are still some discussions on the droplet wetting mechanism^41,42^. In addition, the proposed wetting-transition models lack consistency, making experimentation a must needed step for the superhydrophobic surfaces to be put into practical use. Research groups have mainly focused on the fabrication of superhydrophobic surfaces and on the demonstration of its superhydrophobicity (static and dynamic). Very few groups have worked on the concepts behind droplet wetting or on optimizing the superhydrophobic nature of a surface^43–45^. And also, there are no studies providing a clear comparison of superhydrophobic surfaces on a common scale to enable their design and proper applications. The apparent contact angle and the contact angle hysteresis, the parameters which are commonly reported as a measure of surface’s superhydrophobicity, do not reflect the liquid-pressure stability of the surface. Therefore, there is a need for a parameter capable of both providing a comparison between various superhydrophobic surfaces and explaining the droplet physics.

And also, experimental investigations on the micro-capillary bridges and the contact line depinning during droplet evaporation are rare. Paxton *et al*.^46^ have used an environmental scanning electron microscope (ESEM) to observe the moving contact line of a water droplet at micron length scales. Although, the interaction of capillary-bridging is thoroughly discussed by varying the microfeature spacing and by varying the roughness of the microfeature itself, its effect is not incorporated in the calculations of the vertical adhesion force for depinning of the contact line (due to the difficulty in its estimation). The deviations in the adhesion force measurements observed at large contact line pinned fractions are reasoned to be due to the interaction of capillary-bridging; however, there is an inherent mistake in the conceptualization of the effective pinned fraction (Ф), which is the major reason for those deviations.

Here we propose a new model for explaining the evaporation-triggered wetting transition using a critical force per unit length (FPL) value. This model allows the educated selection and design of the superhydrophobic surfaces. The proposed model is verified by performing droplet evaporation studies on patterned cylindrical and line-shaped microstructures made of poly-perfluorodecylacrylate (pPFDA) coated vertically aligned carbon nanotubes (VA-CNTs) via initiated-chemical vapor deposition (iCVD)^47–54^. And also, we relate the droplet curvature to the conventional beam deflection of solid-mechanics which helps in predicting the type of wetting transition (depinning or touch-down). We avoid the capillary-bridging effect and discuss the lone impact of contact line-fraction on receding contact angles and droplet evaporation modes. This understanding leads to an efficient and experiment-free way of designing and fabricating pressure-stable superhydrophobic surfaces. As a typical example, the spreading of ink on the substrate and its detachment from the stamp can be engineered to increase the speed and the resolution of the flexography printing^47,51,55^.

## Results

### Droplet evaporation studies

#### Varying the micropillar height (H)

**Figure 1:**
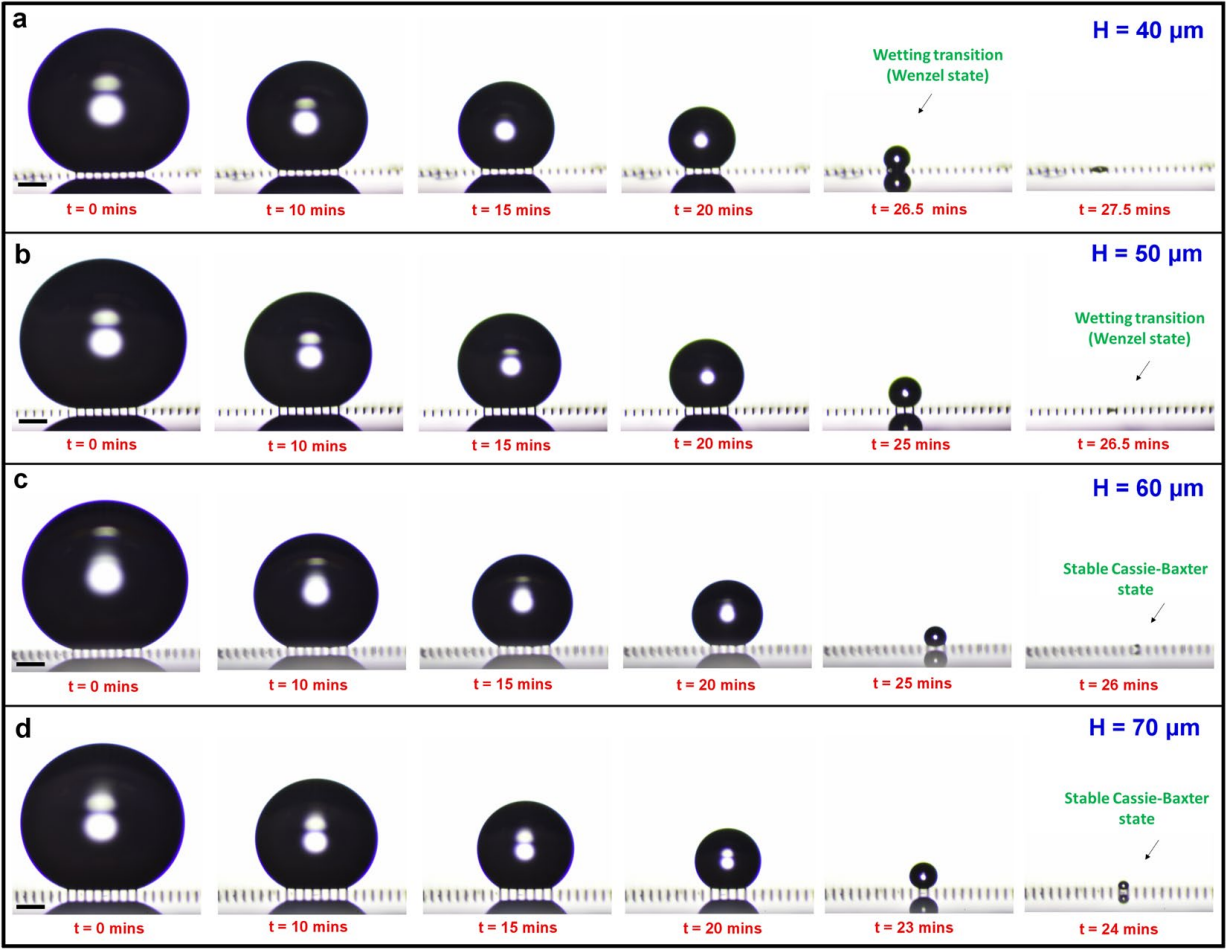
The effect of the micropillar height (H) on the evaporation-triggered wetting transition. Sequential images of an evaporating water droplet (4 μL) on the pPFDA-coated VA-CNT micropillars (radius, r = 10 μm and center-to-center spacing, S = 100 μm) with different heights, H = 40 μm (**a**), 50 μm (**b**), 60 μm (**c**), and 70 μm (**d**). A Cassie-to-Wenzel wetting transition is observed with micropillar heights (H) of 40 μm and 50 μm. A stable droplet evaporation is observed with micropillar heights (H) of 60 μm and 70 μm, even though the Laplace Pressures (P_L_) are higher (1549.2 N/m^2^ and 1058.7 N/m^2^ respectively) than those calculated for the micropillar height (H) of 40 μm (P_L_ = 812.8 N/m^2^). The water droplet exhibited stable wettability (in terms of both apparent contact angle (θ) and prevention of Cassie-to-Wenzel transition during droplet evaporation) with micropillar heights (H) of 60 μm and 70 μm. The scale bar in (**a**,**d**) is 300 μm.

The sample surfaces consisted of poly-(*1 H, 1 H, 2 H, 2H*-perfluorodecylacrylate) (pPFDA) coated nanoporous carbon nanotube (CNT) micropillars^56^ with radius r = 10 μm, center-to-center spacing S = 100 μm, height H varying from 40 μm to 70 μm (see Supplementary Fig. S-1a). A rame-hart automated dispenser was used for the deposition of the water droplets (4 μL) on the sample surfaces. A Nikon (D5500) camera was used to capture the images during the evaporation of the water droplet. The contact angles (θ) and the Laplace pressures (P_L_ = 2*γ*_*L*_/*R*, given by Young’s-Laplace equation^57,58^, where *γ*_*L*_ is the surface tension of the liquid and R is the radius of curvature of the droplet) were plotted during the entire droplet evaporation (see Supplementary Fig. S-1b). Time-lapse images of the evaporating droplets were compared at regular intervals (Fig. 1) for observing the effect of the micropillar height on the droplet evaporation. Cassie-to-Wenzel wetting-transition was observed on samples with micropillar heights of 40 μm and 50 μm, whereas the droplet exhibited a stable Cassie-Baxter state on samples with micropillar heights of 60 μm and 70 μm. As the area-fraction^59^ is low $({\rm{f}}=\frac{\pi {r}^{2}}{{S}^{2}}=0.03)$, we believe that this is a result due tothe droplet attaining a stable state at higher micropillar heights (meeting the critical aspect ratio requirement) as discussed in several research studies^60–63^. The transition from the metastable Cassie-Baxter state to the stable Cassie-Baxter state happens at a critical micropillar height somewhere between 50 μm – 60 μm, which can be determined only through experiments, and is not the focus of this work. Previously, a critical aspect-ratio (height to diameter) of 2 and above^62^ and 3^63^ are reported for attaining the stable hydrophobicity. The micropillar height of H = 60 μm meets both these requirements, which is in accordance with the droplet evaporation experiments. M Lundgren *et al*.^61^ performed molecular dynamics simulations of water droplets with varying pillar heights and concluded that the droplet penetration between the gaps is low at higher pillar heights making contact angles independent of the pillar height. Therefore, it is necessary to ensure that the samples height meet the critical aspect ratio requirement to prevent geometry-induced instability effect on the wettability studies.

Similar trends were observed in the contact angles and the Laplace pressures on all samples (see Supplementary Fig. S-1b). Videos of the droplet evaporation are provided in Supplementary Movies 1–4. Surprisingly, the samples with micropillar heights of 60 μm and 70 μm attained higher Laplace pressure values (1549.2 N/m^2^ and 1058.7 N/m^2^ respectively) than with a height of 40 μm (812.8 N/m^2^) and still prevented the wetting-transition. Therefore, the critical Laplace pressure alone is not a defining parameter for explaining the wetting-transition striking-off all the previous explanations framed on it^30^.

Previous wetting-transition models developed the wetting transition criterion either by using critical Laplace pressure^41^ or by performing force balance^30^ (which is also based on critical Laplace pressure) across the droplet base. It is reported and concluded that the wetting transition is a force balance phenomenon and happens when the downward force due to Laplace pressure exceeds the capillary forces acting in the upward direction^30^. The Laplace pressure at which this condition is met is called the critical Laplace pressure. When a droplet in Cassie-Baxter state attains the critical Laplace pressure, the droplet starts impaling into the micropillars. If the droplet attains the critical Laplace pressure, but depins immediately from the micropillar onto the adjacent micropillar, wetting transition may not happen. The droplet should evaporate in that pinned state (after attaining the critical Laplace pressure) for a considerable duration to allow the droplet movement along the micropillar sidewall, filling the gaps and making it difficult for further depinning. Because, during depinning of TPCL from one micropillar to another, Laplace pressure changes, changing the possibility of the happening of wetting-transition. The Laplace pressure attained during droplet evaporation is determined by the droplet curvature (i.e. the radius of the spherical cap) which is in turn determined by depinning of the three-phase contact line (TPCL). Hence it is very important to understand the droplet evaporation process to explain evaporation-triggered wetting transition. During droplet evaporation, the size of the droplet decreases, changing the droplet curvature (based on the droplet evaporation mode, either constant contact radius (CCR) mode or constant contact angle (CCA) mode). The curvature of the droplet exhibiting CCA mode is greater (or alternatively the droplet radius is smaller) and hence attains higher Laplace pressures when compared to a similar droplet exhibiting CCR mode and is more prone to wetting transition. As mentioned earlier, the mode of evaporation (CCA or CCR) is determine by the TPCL depinning behavior. On superhydrophobic surfaces, the adhesion between the surface and the droplet is low (due to low surface energy and large spacing between the micropillars) enabling the easy depinning of the TPCL, and the evaporating droplet assumes CCA mode of droplet evaporation. The depinning of the droplet and the capillary bridging effect on hydrophobic surfaces is discussed by Paxton *et al*.^46^ and it is concluded that the TPCL depinning is determined by the force balance between interfacial (between surface and liquid) adhesion force along the TPCL and vertical component of the liquid surface tension forces. For superhydrophobic surfaces, the vertical component of the surface tension force (γ_L_Sinθ^app^) is greater enough to overcome the surface adhesion force (determined by γ_SL_). The depinning of the TPCL, and hence the droplet evaporation modes (CCA and CCR) are based on which force (the surface tension force or the adhesion force) being more dominant and for how much duration. Whenever the surface tension force exceeds the interfacial adhesion force, TPCL depins from the micropillar surface and moves on to the adjacent micropillar decreasing the droplet radius (or increasing the droplet curvature) and hence increasing the Laplace pressure. It is also important to mention here that the curvature of the overall droplet and the curvature of the droplet in-between the micropillars is the same, as both these interfaces are experiencing same Laplace pressure. The curvature of the droplet between the micropillars is an important parameter in predicting the touch-down type wetting transition.

**Figure 2:**
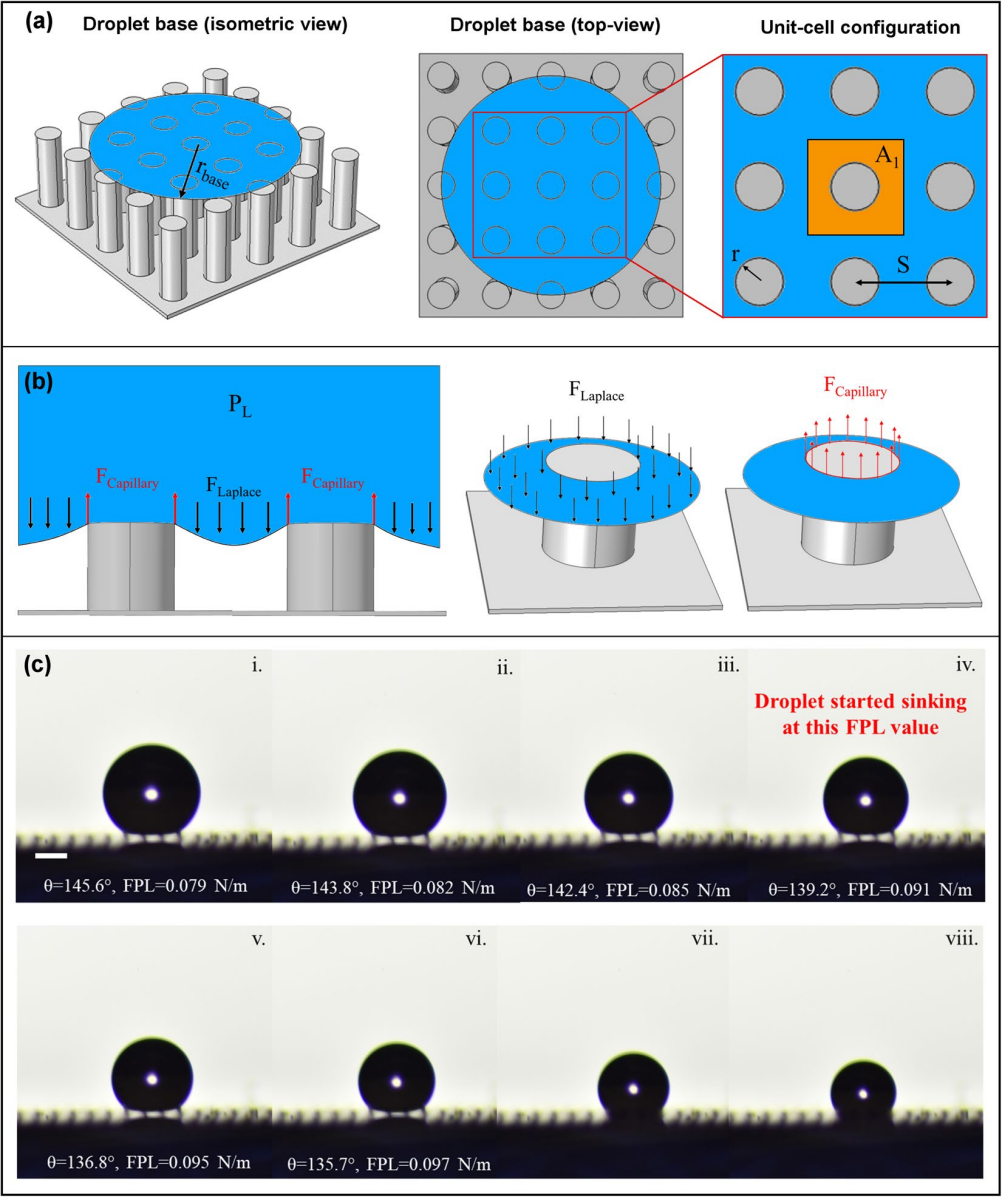
Explaining the wetting-transition using local force balance model. (**a**) Schematic showing the base of a suspending droplet (left), top view of the droplet base (middle), and unit-cell configuration (right). The micropillar in zone A_1_ (unit cell) experiences larger downward force (F_Laplace_) than the peripheral micropillars (see Supplementary Fig. S-2) and hence used for defining the wetting-transition criteria. (**b**) Schematic (side-view) of a droplet curvature between two adjacent micropillars showing the downward Laplace pressure force and upward capillary forces, shown together (left), shown separately (middle & right). The Laplace pressure force also acts on the top of the micropillar (not shown) but cannot affect the wetting-transition phenomenon. (**c**) Shows the determination of the critical force per unit length (FPL) value experimentally. The water droplet started sliding down (depinning) along sidewalls of the inner micropillars when the FPL reached a critical value of 0.090 ± 0.005 N/m. The scale bar in (**c**) is 200 μm.

It is reported that the surfaces with higher area-fraction (i.e. micropillars that are closely placed) are more stable against liquid intrusion pressures and do not exhibit wetting-transition^30^. Alternatively, the critical Laplace pressure causing wetting-transition can be enhanced by increasing the area-fraction or for a given area-fraction the Laplace pressure has to be smaller than the critical Laplace pressure. The higher area-fraction means greater solid-liquid contact area within a unit-cell and hence greater capillary force in the upward direction. This leads to an understanding that the critical Laplace pressure, the load-sharing among the micropillars (or alternatively the solid-liquid contact length in a unit-cell), and the happening of wetting-transition are related to each other. So, the attainment of critical Laplace pressure, the stage of evaporation at attainment (during initial stages or in the final stage), and the duration of the droplet evaporation in that pinned state together determine the happening of wetting-transition. From the time-lapse images (Fig. 1), the sample with a height of 40 μm attained higher Laplace pressure values in the early stages of droplet evaporation, whereas for samples with micropillar heights of 60 μm and 70 μm the droplet attained the same Laplace pressure value almost at the last stage of droplet evaporation. The only difference being the droplet base radius (r_base_) and the number of micropillars beneath it. Most of the wetting-transition models (either based on the energy barrier^29,32,40^ or the force balance^30^) have considered the entire droplet base in their models. However, the droplet is not intelligent to see the whole picture and take care of everything. Similarly, a micropillar can only balance the forces acting on it. It does not even know what is happening on the micropillar next to it. Considering these facts, we develop a model for explaining the wetting-transition using a unit-cell configuration and by dividing the droplet base into different force zones (Fig. 2a and Supplementary Fig. S-2) based on their location beneath the droplet base (A_1_, A_2_, and A_3_). The micropillar in zone A_1_ (unit-cell), which is completely beneath the droplet base, experiences a higher downward force (a force due to the Laplace pressure) because of the larger available area (${F}_{{{\rm{Laplace}}}_{1}}=\,{P}_{{\rm{L}}}\times {A}_{1}$). Similarly, zones under A_2_ and A_3_ also experience downward Laplace pressure force such that $\,{F}_{{{\rm{Laplace}}}_{3}} < {F}_{{{\rm{Laplace}}}_{2}} < {F}_{{{\rm{Laplace}}}_{1}}$. For, the micropillars arranged at larger center-to-center spacing (i.e. lower area-fraction), the unit-cell area is greater and hence experience a higher downward force and if higher enough, enable the droplet to exhibit a Wenzel droplet state immediately after the droplet deposition. The droplet starts sliding (depinning) along the micropillar side-wall when the downward Laplace pressure force (F_Laplace_) exceeds the maximum upward capillary force (F_Capillary_). The capillary force acts along the circumference of the micropillar along the TPCL in the upward direction (Fig. 2b). The higher the perimeter (P) of the micropillar, the higher is the upward capillary force and better is the Cassie-Baxter state stability. Here, we introduce the term “critical force per unit length (FPL) value” for estimating the maximum upward capillary force exerted by the micropillar. The previous wetting transition models^30,45^ focused on the droplet, determining the critical Laplace pressure. Whereas here, we focused on the micropillar itself, determining its critical FPL value.

1 $${{\rm{F}}}_{{\rm{Laplace}}}={P}_{{\rm{L}}}\times A=P\times FPL$$

and

2 $${{\rm{F}}}_{{\rm{Capillary}}}=P\times FP{L}_{{\rm{Critical}}}$$

where *P* is the perimeter of the micropillar, A is the unit cell area (shown as *A*_1_ in Fig. 2) given by *A* = *S*^2^−*πr*^2^ (see Fig. 2a). Based on the surface chemistry every material has its own critical FPL value equal to *γ*_L_*Cosθ*^adv^ (details of which are discussed later), where *γ*_L_is the surface tension of the liquid and *θ*^adv^ is the advancing contact angle of the liquid droplet on the smooth surface. The higher the hydrophobicity of the material (i.e. higher the advancing contact angle), the higher its critical FPL value. By tuning the perimeter of the micropillar (P) and the surface chemistry (critical FPL value), the upward capillary force (F_Capillary_) can be tuned as required for preventing the wetting-transition. The condition F_Laplace_ > F_Capillary_, or alternatively FPL > FPL_Critical_, is met on the inner micropillars first. The same was observed in the droplet evaporation experiments with more penetrations along the inner micropillars than the peripheral micropillars (Fig. 2c). The inherent re-entrant shape of the CNT micropillar (Supplementary Fig. S-3) make the theoretical calculation of critical FPL value difficult. From the experiments, the critical FPL value of the pPFDA-coated CNT micropillar is found out to be = 0.090 ± 0.005 N/m.

**Figure 3:**
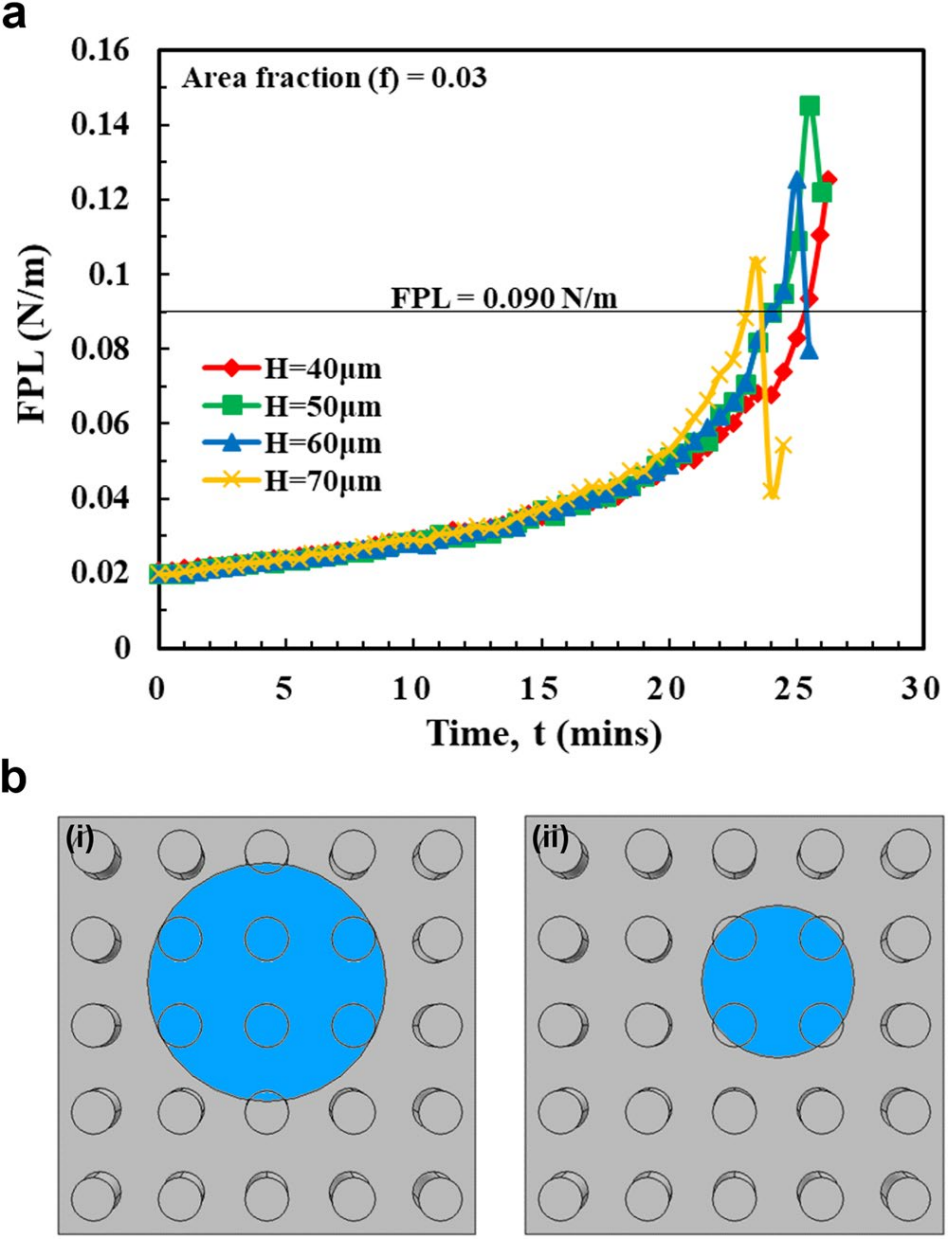
Explaining wetting-transition using critical force per unit length (FPL) value. (**a**) Plot showing the variation in the FPL value during the evaporation of a 4 μL water droplet on the pPFDA-coated VA-CNT micropillars (radius, r = 10 μm, center-to-center spacing, S = 100 μm, and area-fraction, f = 0.03) with different heights (H) of 40 μm, 50 μm, 60 μm and 70 μm. The FPL value exceeded the critical FPL value of 0.090 N/m on samples with all micropillar heights, but it remained above 0.090 N/m on samples with micropillar heights of 40 μm and 50 μm, whereas it quickly dropped to below 0.090 N/m on samples with the pillar heights of 60 μm and 70 μm, preventing the wetting-transition. (**b**) Schematics showing the droplet base (top-view) during the last stages of droplet evaporation. The downward force due to Laplace pressure in a unit-cell that is previously balanced by a single micropillar in (i) is now balanced by four micropillars in (ii), reducing the FPL value drastically.

To verify the local force balance model, we calculate the FPL values for the corresponding Laplace pressures using Equation (1) (Fig. 3a). On samples with micropillar heights of 40 μm and 50 μm, the FPL values exceeded the critical FPL value and maintained over it for a considerable period of time; this allows droplet movement along the side-wall of the micropillar causing wetting-transition. Whereas, on samples with micropillar heights of 60 μm and 70 μm, the FPL values exceeded the critical FPL value but quickly dropped below it resisting the droplet movement and hence preventing the wetting-transition. This change in the FPL trend is due to a change in the force distribution (or load-sharing) across the micropillars. With micropillar heights of 60 μm and 70 μm, at the instant when the FPL value exceeded the critical FPL value, the droplet is in the last stage of evaporation with four micropillars beneath it. The area that is previously balanced by a single micropillar is now balanced by four micropillars (Fig. 3b) reducing the FPL value drastically. The effective P in Eq. (1) changes to 4 P (theoretically). Assuming that the droplet base is still circular, a conservative value of 2.5 P is used in the calculations.

#### Varying the area-fraction (f)

**Figure 4:**
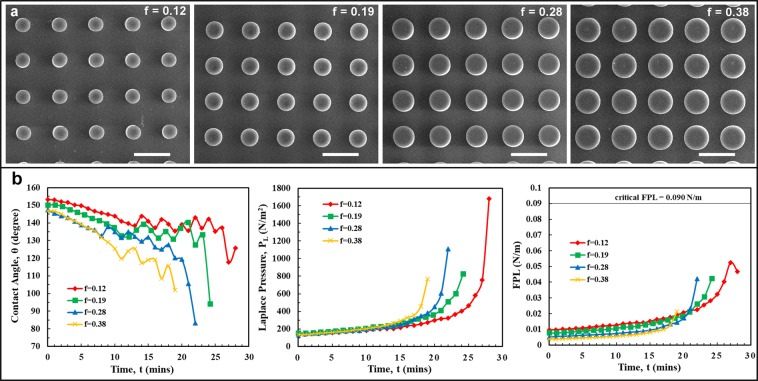
The effect of the area-fraction (f) on the wetting transition. (**a**) Top-view SEM images of the pPFDA-coated VA-CNT micropillar samples (height, H = 90 µm and center-to-center spacing, S = 100 μm) with area-fractions (f) of 0.12, 0.19, 0.28, and 0.38 (left to right) for micropillar radius (r) of 20 µm, 25 µm, 30 µm, and 35 µm respectively. The scale bar is 100 µm. (**b**) Plots showing the variation in the contact angle, θ (left panel), Laplace pressure, *P*_L_ (middle panel) and force per unit length, FPL (right panel) during the evaporation of a 4 μL water droplet. The calculated FPL values are much lower than the critical FPL value of 0.090 N/m for all the area-fractions (f) further verifying the local force balance model in prediction of wetting-transition.

Samples consisted of pPFDA-coated CNTs with micropillars heights of H = 90 μm, center-to-center spacing, S = 100 μm, and radius, r varying from 20 μm to 35 μm, resulting in the area-fractions (f) varying from 0.12 to 0.38 (Fig. 4a). The micropillar height was selectively chosen to be 90 μm to avoid the geometry-induced instability effects. The contact angles, Laplace pressures, and the corresponding FPL values were plotted (Fig. 4b). Videos of the droplet evaporation are provided in Supplementary Movies 5–8. Wetting-transition (either depinning or touch-down) was not observed in any of the samples. As discussed in the previous section, the micropillar height of 90 μm is long enough to prevent the touch-down type wetting-transition on the above samples. The calculated FPL values attained during droplet evaporation were much lower than the critical FPL value, preventing the depinning of the TPCL along the micropillar side-wall and therefore preventing the wetting transition. Lower Laplace pressure values and hence lower FPL values were observed at higher area-fractions, reducing the possibility of wetting-transition. Two important conclusions can be drawn from these observations. First, if the wetting-transition does not happen at a given area-fraction, it will not happen at subsequent higher area-fractions (that everything else remain the same). Second, the geometry of the microstructures can be determined using the critical FPL value and the local force balance model to meet the pressure-stability requirements. Alternatively, for a given value of liquid pressure we can predict the wetting-transition and the type of wetting-transition (depinning or touch-down).

#### Varying the microstructure cross-sectional shape

**Figure 5:**
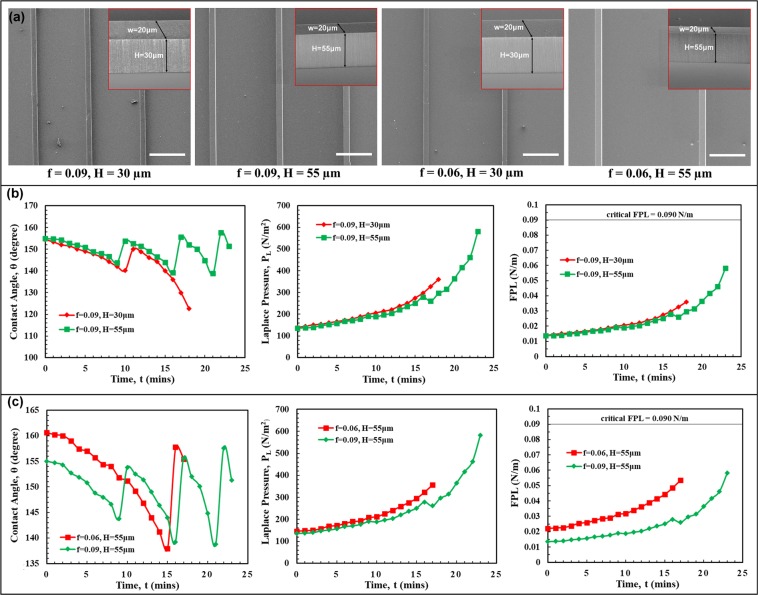
Verification of the local force balance model using line-shaped microstructures. (**a**) Top-view SEM images of the line-shaped microstructures with varying heights (H) and area-fractions (f). Insets in (**a**) shows zoomed and tilted SEM images. The scale bar in (**a**) is 150 μm. (**b**,**c**) Plots showing the variation in the contact angle, θ (left panel), Laplace pressure, *P*_L_ (middle panel) and FPL (right panel) during the evaporation of a 4 μL water droplet with varying micropillar heights and area-fractions, respectively. **(b)** The droplet evaporated in a similar manner with the heights of H = 30 μm and H = 50μm, except that the wetting transition (touch-down) has occurred early with H = 30 μm than with H = 55 μm. **(c)** Inspite of having similar trends in the Laplace pressure (P_L_), the FPL is higher with f = 0.06 than with f = 0.09. As the area-fraction (f) decreases, the center-to-center spacing (S) between the microstructures increases and hence the FPL increases. Therefore, the microstructures with larger center-to-center spacing are more prone to the wetting-transition (touch-down). Wetting transition (touch-down) is observed with both the area-fractions. A Wenzel droplet state is observed with f = 0.06 and H = 30 μm right from the beginning. No depinning is observed with any of heights (H) and area-fractions (f) as the FPL is lower than the critical FPL value of 0.090 ± 0.005 N/m, confirming the microstructure cross-sectional shape independency of the critical FPL value.

We performed the droplet evaporation studies using line-shaped microstructures to verify dependence of our model to cross-sectional shape of microstructure. Samples of line-shaped pPFDA-coated CNT microstructures were prepared with varying heights and center-to-center spacing (Fig. 5a). The contact angles, Laplace pressures, and the corresponding FPL values were plotted for the duration of the entire droplet evaporation. Separate plots were made to clearly distinguish the effects of pillar height (Fig. 5b) and area-fraction (Fig. 5c). Videos of the droplet evaporation are provided in Supplementary Movies 9–11. Sample with area-fraction of f = 0.06 and micro-structure height of H = 30 μm exhibited an initial Wenzel droplet state due to liquid curvature touching the bottom substrate. The effect of center-to-center spacing on the FPL values was apparent in Fig. 5c (right). Though the Laplace pressure trends were similar, the calculated FPL values are higher for larger spacing due to larger available area as explained earlier. Touch-down type of wetting-transition was observed on all samples. This was expected as the area-fractions (f = 0.09 and f = 0.06) were low and the microstructure heights (H = 30 μm and H = 55 μm) were small. The FPL values were lower than the critical FPL (0.090 N/m) value throughout the droplet evaporation, eliminating the chance of depinning. These observations further validate the concept of the critical FPL value and the local force balance model. From these observations, we propose that the critical FPL value is related only to the material and the liquid and is independent of the microstructure cross-sectional shape. It is important to note that the concept of the critical FPL value is established using cylindrical and line-shaped microstructures on which the force distribution (or load-sharing) is more uniform and are good for comparison purpose, unlike the square-shaped micropillars where the sharp corners experience higher downward force than the edges (Supplementary Information, Fig. S-5) similar to the stress-concentration in the solid-mechanics.

**Figure 6:**
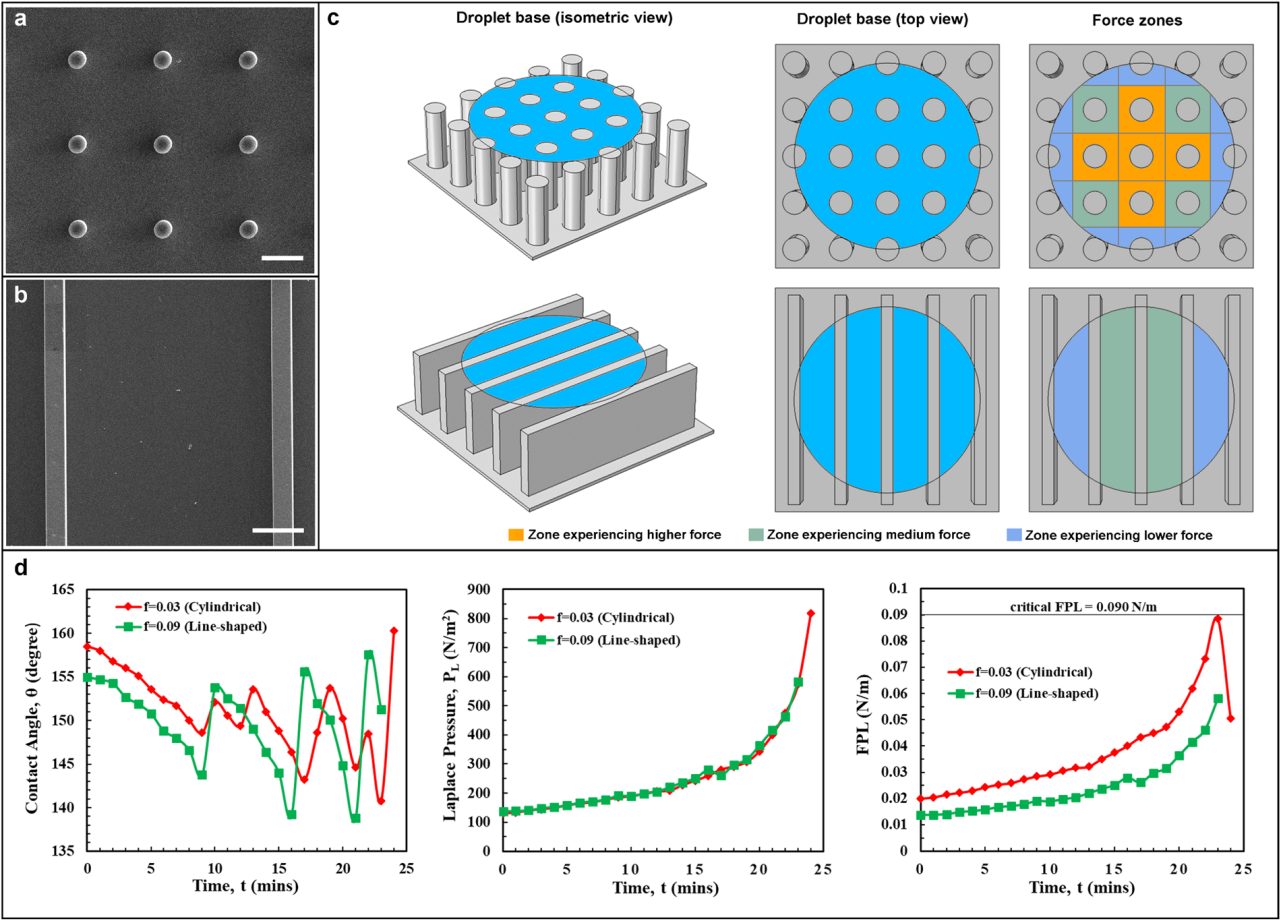
Comparison of stability to liquid pressure between cylindrical and line-shaped microstructures. (**a**) and (**b**) Shows the SEM images of the cylindrical (f = 0.03) and line-shaped (f = 0.09) pPFDA-coated VA-CNT microstructures, respectively. (**c**) A schematic showing a typical droplet base (droplet curvature is not shown) and the different force zones (high, medium, and low are relative, just shown for better understanding) with the cylindrical and line-shaped microstructures. (**d**) Plots showing the variation in the contact angle, θ (left panel), Laplace pressure, P_L_ (middle panel) and FPL (right panel) during the evaporation of a 4 μL water droplet on the cylindrical (f = 0.03) and line-shaped microstructures (f = 0.09). Though the trends of the Laplace pressure (P_L_) are the same, the FPL values with cylindrical microstructures are higher than the line-shaped microstructures as the cylindrical microstructures have smaller solid-liquid contact length for a given center-to-center spacing (S). Alternatively, line-shaped microstructures offer enhanced solid-liquid contact length for a given center-to-center spacing (S) and hence have lower FPL values and are more stable to liquid pressure than the cylindrical microstructures. The scale bar in (**a**,**b**) is 50 μm.

The dynamic pressure-stability of the cylindrical and line-shaped microstructures (Fig. 6) is also studied. The samples (Fig. 6a,b) with similar Laplace pressure trends (Fig. 6d, middle) were chosen for comparison. From the plot of FPL value (Fig. 6d, right), it was observed that the pressure-stability of the line-shaped microstructures (center-to-center spacing = 200 μm) is higher than the cylindrical microstructures (center-to-center spacing = 100 μm). This may be due to the fact that line-shaped microstructures offer higher solid-liquid contact lengths than the cylindrical microstructures (Fig. 6c) for a given area. In other words, the distribution of the force (or the load-sharing) is more with the line-shaped microstructures than with the cylindrical microstructures. The same was reflected in the droplet impact experiments. Water droplet (8 μL) was impacted from a height of 2 cm on both the samples. The droplet bounced back multiple times on the line-shaped microstructures (Supplementary Movie 12), whereas on the cylindrical micropillars the droplet infiltrated and wetted the surface completely (Supplementary Movie 13).

## Discussion

### Critical FPL Value

We introduce the concept of critical force per unit length (FPL) value and use the local force balance model to explain the wetting-transition mechanism. C. W. Extrand used a similar approach to explain the water repellency of the lotus leaf^45,64^. Those studies have focused on the determination of critical Laplace pressure using a unit cell model. But, the critical Laplace pressure changes with the microstructure geometry and shape, and cannot be generalized. As discussed in the previous sections, the timing of the droplet attaining the critical Laplace pressure is also important as the force distribution in a unit-cell is dependent on the number of microstructures included in it. And also, some wetting-transition models used apparent contact angle while calculating the upward capillary force instead of advancing contact angle^30^. Here, we used the advancing contact angle (*θ*^adv^) in the critical FPL value calculations for compensating the line-tension^65,66^ and pinning force^33^ effects. Since those quantities (line tension and pinning force) are very small in magnitude and as there are no accurate methods and equipment available for their measurement, using advancing contact angle will compensate those effects to some extent.

The term “force per unit length (FPL)” reminds the liquid surface tension (or maybe the mechanical spring constant). Also, from the definition of critical FPL value (FPL_Critical_ = *γ*_L_*Cosθ*^adv^), it appears to be true. From the above relation, the maximum value of the FPL_Critical_ is limited by the surface tension of the liquid *γ*_L_ due to mathematical constraints. With water, the maximum value is approximately 0.072 ± 0.005 N/m. But, the critical FPL value of the pPFDA-coated CNTs is found out to be 0.090 ± 0.005 N/m, which is greater than the maximum possible value. This is due to the inherent re-entrant shape of the CNT micropillar (Supplementary Fig. S-3). Researchers have used re-entrant shaped microstructures for enhancing the superhydrophobic and omniphobic properties of the surfaces^43,67^. For a micropillar with shape angle (*φ*) (Supplementary Fig. S-4), the formula for the critical FPL value changes to FPL_Critical_ = *γ*_L_*Cos*(90 + *φ* − *θ*^adv^). Almost every study in the superhydrophobic field limit the maximum upward capillary force (with either vertical or re-entrant shaped micropillars) to the liquid surface tension^30,45^. However, the effect of the re-entrant shape can be clearly visualized with the critical FPL value which can be larger than the liquid surface tension (ranging from *γ*_L_ to 2 *γ*_L_). It is always good to find out the critical FPL value experimentally (even for smooth and vertical microstructures) as it provides the final and direct value incorporating the shape and size effects of the microstructure. Overall, the critical FPL value ranges from 0 to 2 *γ*_L_ providing a common scale for all the microstructure shapes and materials. For vertical microstructures, having uniform force distribution along their circumference (like the cylindrical and line-shaped), it is referred to as “critical FPL value of the material”. For microstructures with non-uniform force distribution (like square and polygon shaped), and with a shape angle (*φ*), the critical FPL value is referred to as “critical FPL value of the microstructure”.

### Stiffness of the Liquid

**Figure 7:**
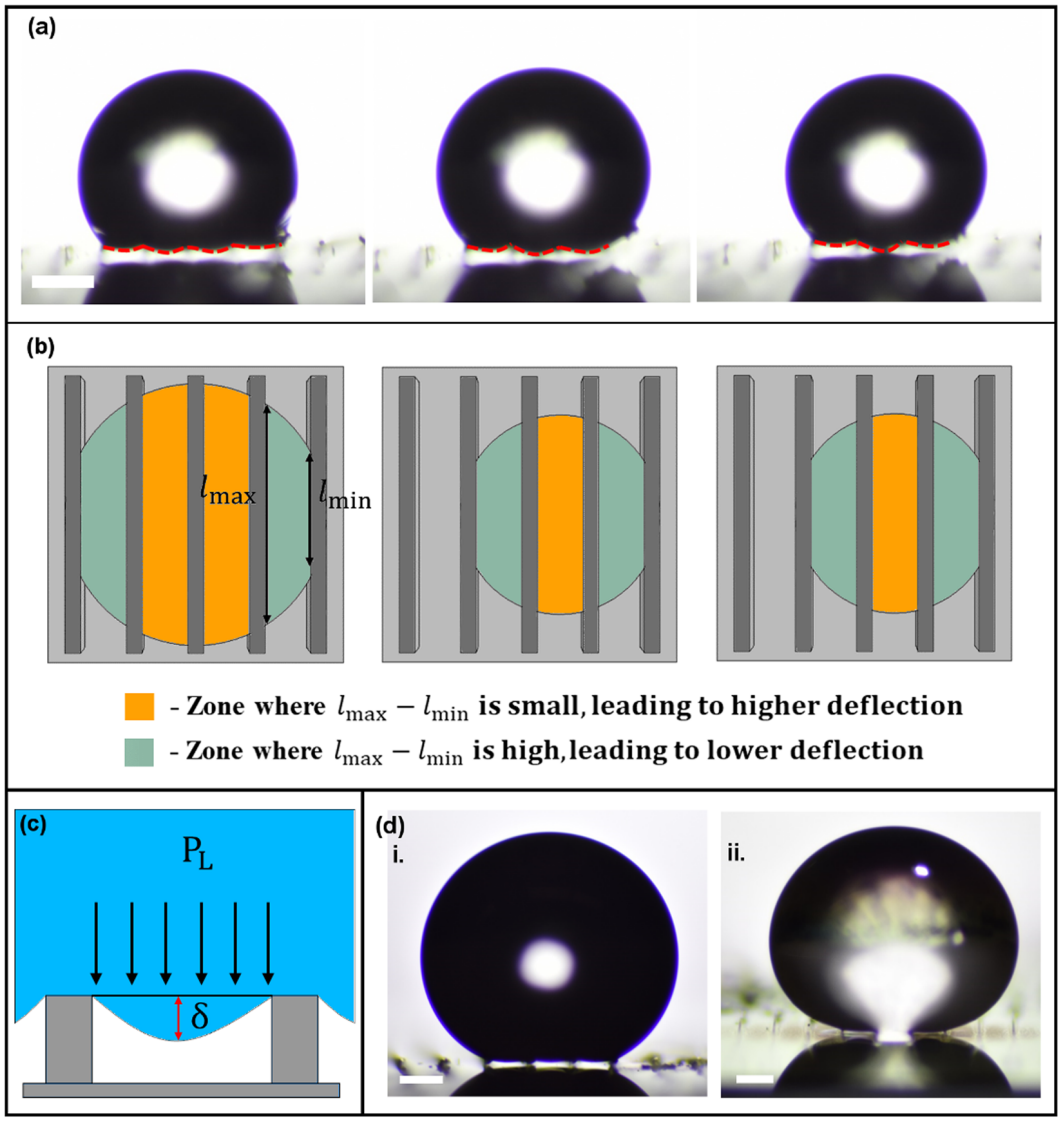
Similarities between droplet curvature (deflection, δ) and beam deflection. (**a**) Optical images showing the uneven droplet curvature (deflection, δ) across the droplet base. The scale bar is 300 μm. (**b**) Schematics showing the force zones across the droplet base and the difference in the solid-liquid contact lengths (l_max_-l_min_) across the zones. Similar to the deflection of the beam under a uniform load, the deflection (δ) will be higher when the difference in the solid-liquid contact lengths (l_max_-l_min_) is lower. The l_max_-l_min_ is minimal at the center of the droplet causing higher deflection (δ). The same is reflected in the droplet curvature. (**c**) Schematic showing the deflection, δ between two micropillars of a suspended droplet. (**d**) Images of the as-deposited droplets of (**i**) water (*γ*_Water_ = 72.5 ± 0.3 mN/m), and (**ii**) ethyleneglycol (*γ*_EG_ = 47.7 ± 0.2 mN/m). The ethyleneglycol droplet touched the substrate stating that the liquid stiffness of ethyleneglycol is lesser than water. The scale bar is 200 μm.

Using the concept of critical FPL value, we explain the depinning type of wetting-transition. When the spacing between the micropillars is large, as is the case for line-shaped microstructures, the wetting-transition cannot be explained using the critical FPL value alone. The curvature of the droplet may touch the bottom surface even before the force due to Laplace pressure reaches the critical FPL value, leading to a touch-down type of wetting-transition. For the line-shaped microstructures, the FPL values attained during the droplet evaporation period were lower than the critical FPL value (Fig. 5) and the wetting-transition happened due to the droplet curvature (hereafter referred to as deflection, δ) touching the bottom substrate or becoming equal to the microstructure height (H). And also, the deflection is not uniform across the droplet base (Fig. 7a). This non-uniformity in the droplet deflection may not be due to the differences in the hydrostatic pressure across the droplet base, as its magnitude is very small when compared to the Laplace pressure. The only difference is the force distribution across the droplet base. The center zones experience higher force than the outer zones due to the difference in the contact lengths (l_max_-l_min_) across it (Fig. 7b). This reminds the conventional solid-mechanics beam deflection. We aim to relate the deflection, δ (Fig. 7c) with the Laplace pressure (P_L_) and the edge-to-edge gap (d) between two adjacent micropillars using the following relation:

3 $$\delta =\frac{2{P}_{{\rm{L}}}\times {d}^{2}}{{k}_{{\rm{L}}}}$$

where *k*_L_ (N/m) is the “stiffness” of the liquid similar to the mechanical spring constant. For the cylindrical microstructures the deflection relation changes to:

4 $$\delta =\frac{{P}_{{\rm{L}}}\times {d}^{2}}{{k}_{{\rm{L}}}}$$

This is not because of the difference in the microstructure shape, it is due to the shift in the deflection mechanism from 1D (line-shaped microstructures) to 2D (cylindrical microstructures). From the experiments, *k*_Water_ = 1 N/m for both the line-shaped and cylindrical microstructures (Supplementary Fig. S-6a,b). We propose that *k*_L_ < 1 N/m for liquids whose surface tension is lesser than the surface tension of water, and *k*_L_ > 1 N/m for liquids whose surface tension is greater than the surface tension of water (Fig. 7d).

### Contact Line-Fraction

The wetting-transition (either by depinning or touch-down) is based on the Laplace pressure (*P*_L_) attained during the droplet evaporation, which is determined by the droplet evaporation modes (constant contact radius, CCR and constant contact angle, CCA). Here, we look into the fundamentals of the droplet evaporation physics to figure out the mode of droplet evaporation, which enables the prediction of the attainable Laplace pressure (*P*_L_).

Although the Cassie-Baxter equation is used extensively in the superhydrophobic field, there are doubts about its validity and applicability^33,41,44^. The Cassie-Baxter equation^16,59^ provides the apparent static contact angle using area-fractions as mentioned below.

5 $$Cos{\theta }^{CB}={f}_{SL}Cos{\theta }^{SL}+{f}_{LA}Cos{\theta }^{LA}$$

where *θ*^*CB*^, *θ*^*SL*^ and *θ*^*LA*^ are the Cassie-Baxter contact angle, contact angle on smooth surface and contact angle with air respectively. The area-fractions, solid-liquid (*f*_*SL*_) and the liquid-air (*f*_*LA*_) area fractions are such that, *f*_*SL*_ + *f*_*LA*_ = 1. Cassie-Baxter equation cannot explain the apparent contact angles during droplet evaporation and the droplet evaporation modes (CCR and CCA). Alternatively research groups studied Pease’s equation^68^ which is based on the three phase contact line (TPCL) pinning.

6 $${W}_{SL}={W}_{p}{L}_{p}+{W}_{np}{L}_{np}$$

*W*_*SL*_ is the work of adhesion along the total length of the TPCL, *W*_*p*_ and *W*_*np*_ are the work of adhesion of the polar and non-polar groups with lengths *L*_*p*_ and *L*_*np*_, respectively. For the solid-liquid-air configuration, the Pease’s equation becomes:

7 $${W}_{TPCL}={W}_{SL}{L}_{SL}+{W}_{LA}{L}_{LA}$$

where *W*_*TPCL*_ is the total work of adhesion, and *W*_*SL*_ and *W*_*LA*_ are the polar (solid-liquid) and non-polar (liquid-air) works of the adhesion corresponding to the contact lengths *L*_*SL*_ and *L*_*LA*_ respectively. The total length of the TPCL is *L*_*TPCL*_ such that *L*_*TPCL*_ = *L*_*SL*_ + *L*_*LA*_. By applying Young-Dupre equation^69^ for the works of adhesion Equation (7) can be written as:

8 $${\gamma }_{L}{L}_{TPCL}(1+Cos{\theta }^{P})={\gamma }_{L}{L}_{SL}(1+Cos{\theta }^{SL})+{\gamma }_{L}{L}_{LA}\,(1+Cos{\theta }^{LA})$$

where *θ*^*P*^, *θ*^*SL*^ and *θ*^*LA*^ are the apparent contact angle on the patterned pillars, contact angle on smooth solid surface, and contact angle with air respectively and *γ*_*SL*_, *γ*_*LA*_ are the solid-liquid, and liquid-air interfacial surface tensions respectively.

Let $\frac{{L}_{SL}}{{L}_{TPCL}}={L}_{1}$ and $\frac{{L}_{LA}}{{L}_{TPCL}}={L}_{2}$, and dividing (8) by *L*_*TPCL*_;

9 $${\gamma }_{L}\,(1+Cos{\theta }^{P})={\gamma }_{L}{L}_{1}\,(1+Cos{\theta }^{SL})+{\gamma }_{L}{L}_{2}(1+Cos{\theta }^{LA})$$

Since, *L*_*TPCL*_ = *L*_*SL*_ + *L*_*LA*_, *L*_1_ + *L*_2_ = 1 and substituting *L*_2_ = 1 − *L*_1_ in (9);

10 $${\gamma }_{L}+{\gamma }_{L}Cos{\theta }^{P}={\gamma }_{L}{L}_{1}+{\gamma }_{L}{L}_{1}Cos{\theta }^{SL}+{\gamma }_{L}(1-{L}_{1})+{\gamma }_{L}{L}_{2}Cos{\theta }^{LA}$$

11 $${\gamma }_{L}+{\gamma }_{L}Cos{\theta }^{P}={\gamma }_{L}{L}_{1}+{\gamma }_{L}{L}_{1}Cos{\theta }^{SL}+{\gamma }_{L}-{\gamma }_{L}{L}_{1}+{\gamma }_{L}{L}_{2}Cos{\theta }^{LA}$$

12 $${\gamma }_{L}Cos{\theta }^{P}={\gamma }_{L}{L}_{1}Cos{\theta }^{SL}+{\gamma }_{L}{L}_{2}Cos{\theta }^{LA}$$

After cancelling out the *γ*_*L*_ throughout in (12);

13 $$Cos{\theta }^{P}={L}_{1}Cos{\theta }^{SL}+{L}_{2}Cos{\theta }^{LA}$$

Equations (13) and (5) look similar, but instead of *f*_*SL*_ and *f*_*SA*_ there are *L*_1_ and *L*_2_. We aimed to bring out the similarities between the contact line-fractions (*L*_1_ and *L*_2_) of the Pease’s equation and the area-fractions (*f*_*SL*_and *f*_*LA*_) of the Cassie-Baxter equation. Using computer software (AutoCAD) the line-fractions *L*_1_ and *L*_2_ are measured for different micropillar arrangements (Supplementary Fig. S-7) and found that the values of the line-fractions *L*_1_ and *L*_2_ are very close to area fractions *f*_*SL *_and *f*_*LA*_ (Supplementary Table S-1). Since the measurement of *L*_1_ and *L*_2_ is difficult, *f*_*SL*_and *f*_*LA*_ can be used to get an estimate of the initial droplet apparent contact angle. The contact line-fraction *L*_1_ is also referred as the effective pinned fraction (Φ), given by ${\rm{\Phi }}=\frac{P}{\tau }$^46^, where P is the perimeter of each micropillar and τ is the micropillar pitch. A comparison is made between *f*_*SL*_, *L*_1_, and ${\rm{\Phi }}$ (Supplementary Table S-1) only to find that Φ is much higher than the *L*_1_ and *f*_*SL*_. Therefore, we conclude that the apparent contact angle is based on the balance between the works of adhesion along the TPCL. For a heterogeneous surface, the contact angle equation is given by:

14 $$Cos{\theta }^{app}={\sum }_{i=1}^{n}{L}_{i}Cos{\theta }^{i}$$

where, *θ*^*app *^is the apparent contact angle, *L*_*i*_ and *θ*^*i*^ are the contact line-fraction and the apparent contact angle of each heterogeneity, and ‘n’ represents the total number of heterogeneities.

Area-fraction (solid-liquid contact area) is calculated for both the line-shaped and the cylindrical microstructures (Supplementary Fig. S-8a). Surprisingly, the calculated area-fraction on the line-shaped microstructures differed from the theoretical value by more than 100 percent due to the higher wetted area at the edges of the droplet (Supplementary Fig. S-8b). This is again a major drawback of the Cassie-Baxter equation.

**Figure 8:**
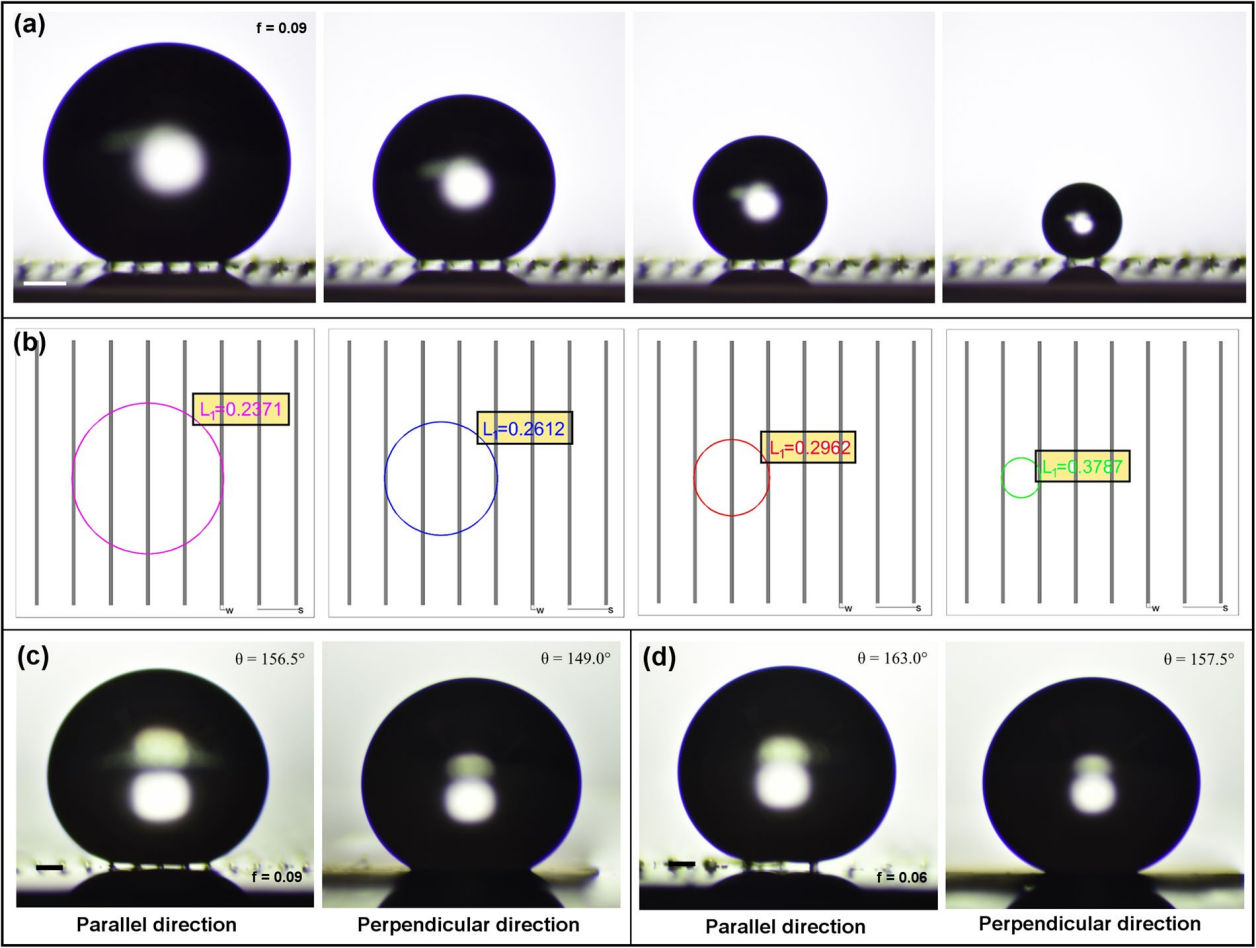
The variation in the contact line-fraction during droplet evaporation. (**a**) Sequential optical images captured during the evaporation of a 4 μL water droplet on the line-shaped microstructures (area fraction, f = 0.09). The scale bar is 300 μm. (**b**) Shows the calculated line-fraction (L_1_) based on the droplet position. Unlike area-fraction (f) which is a constant value, the line-fraction (L_1_) changes during the droplet evaporation effecting the receding contact angles and the droplet evaporation modes. (**c**,**d**) Shows the optical images of as-deposited water droplet (4 µL) on line-shaped microstructures with area fraction, f = 0.09 and 0.06 respectively. The droplet exhibited higher contact angles in parallel direction than in the perpendicular direction for both f = 0.09 and f = 0.06, which supports the proposed contact angle equation using line fractions (see Eq. 13). The scale bar is 200 µm.

**Figure 9:**
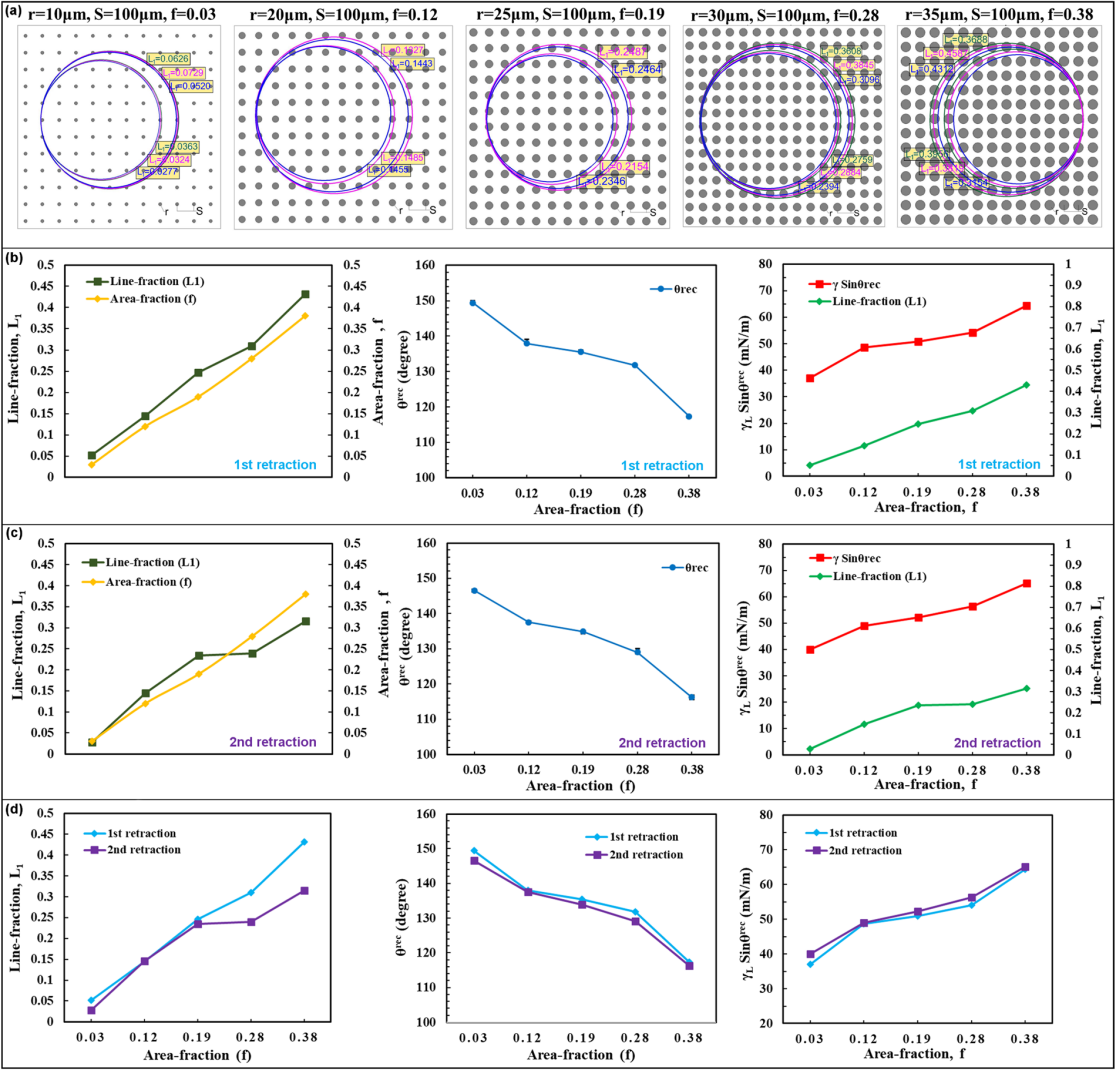
Relation between the receding contact angles and the contact-line fractions. (**a)** AutoCAD drawings showing the calculated contact line-fractions for the successive retractions (1^st^ and 2^nd^) during droplet evaporation on samples with varying area-fraction. Plots showing the variation in the receding contact angle (θ^rec^) and the contact line-fraction (L_1_) with area-fraction (f) for 1^st^ (**b**) and 2^nd^ (**c**) retraction. The receding contact angles decreased with the increasing contact line-fractions requiring higher vertical capillary force (*γ*_L_*Sinθ*^rec^) for TPCL depinning. (**d**) Plots showing the side-by-side comparison of the contact line-fractions and receding contact angles for the 1^st^ and 2^nd^ retractions. The receding contact angles are comparable for the first two retractions.

**Figure 10:**
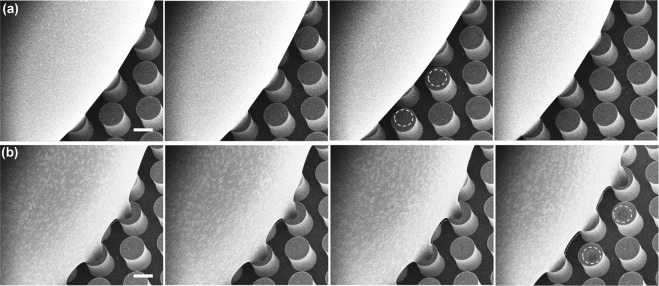
Effect of capillary-bridging on TPCL depinning. Sequential ESEM images (tilt angle = 25°) showing the droplet retraction during initial (**a**) and latter (**b**) stages of evaporation. During initial stages of droplet evaporation capillary-bridging is minimal and surface adhesion and depinning are a sole effect of contact-line fraction. The droplet retracted leaving a small trace of droplet which evaporated immediately (white dotted circle). In the latter stages, the droplet retracted from the micropillars leaving a big micro droplet (white dotted circle) on top of the micropillar due to strong capillary-bridging. The scale bar in (**a**,**b**) is 100 μm.

Unlike the area-fraction (which is a fixed value), the contact line-fraction continues changing during the droplet evaporation. For simplicity, the droplet evaporation is first studied on line-shaped microstructures of width w = 20 μm, center-to-center spacing S = 220 μm (Fig. 8). The contact line-fractions (Fig. 8b) are calculated for the intermittent droplet states to examine its relation with the apparent and receding contact angles. The trends of the measured contact angles from the droplet evaporation experiments are in good agreement with the calculated contact line-fractions (See Supplementary Table S-2). And also, anisotropic behavior is observed in the contact angle values measured in directions parallel and perpendicular to the orientation of the line-shaped microstructures (Fig. 8c,d). On samples with f = 0.06 and f = 0.09, the contact angle values measured in parallel direction are higher (by approximately 6°) than that of the contact angle values measured in perpendicular direction. We propose that this isdue to the differences between the contact line-fractions in the parallel and perpendicular directions. Next, the droplet evaporation is studied on cylindrical micro-structures by varying the area-fraction (Fig. 9). Prediction of the exact position of the droplet is much difficult on cylindrical microstructures than on the line-shaped microstructures. We verify the assumed droplet positions (Fig. 9a) by comparing the calculated contact-line fractions with the theoretical area-fractions. The calculated contact line-fractions are close to the theoretical area-fraction (Fig. 9b,c, left) validating the droplet positions. The receding contact angle values decreased for higher contact-line fractions (Fig. 9b,c, right). This is due to an increase in the adhesion force at higher line-fractions increasing the vertical capillary force required for TPCL depinning^46^. In addition, the receding contact angle values for first two retractions are very close (Fig. 9d, middle). This may be due to weak capillary bridging and similar contact line-fractions. From the environmental scanning electron microscope (ESEM) studies it was noticed that the capillary bridging is minimal during the initial stages of droplet evaporation. Initially, the droplet retracted from the micropillars leaving very small traces of droplet which evaporated immediately (Fig. 10a, Supplementary Movie 14), whereas in the later stages the droplet retracted leaving a micro-droplet (white dotted circle) on the top of the micropillar (Fig. 10b, Supplementary Movie 15). This is due to the strong capillary bridging and necking of the droplet (Fig. 10b). From these observations, we conclude that the adhesion of the droplet along the TPCL is strongly affected by the capillary bridging. Therefore, we restricted ourselves to the first two retractions to distinguish the effects due to contact line-fraction and capillary bridging.

The droplet evaporation modes (CCA and CCR) and the observed stick-slip mechanism during droplet evaporation are determined by the work of adhesion along the TPCL. For lower contact line-fractions the work of adhesion is small and the droplet exhibits larger apparent contact angles and an initial CCA mode of evaporation (Supplementary Fig. S-9a). At higher contact line-fractions, the work of adhesion is greater requiring larger vertical capillary forces for depinning, and hence decreasing the receding contact angle, leading to a CCR mode of droplet evaporation (Supplementary Fig. S-9b,c). The observed stick-slip mechanism during the droplet evaporation is due to the difference between the intermittent apparent contact angle and the receding contact angle for a given droplet state (∆θ). The droplet exhibited CCA mode of evaporation when the ∆θ value is small and shifted to CCR mode for larger ∆θ values. It is important to discuss the effect of micro and nano scale roughness on ∆θ and the wetting-transition. The adhesion along the TPCL can be decreased by multiple folds via incorporating dual (micro/nano) scale roughness for attaining CCA mode, but the wetting-transition is a microstructure edge phenomenon. The intrinsic hydrophobicity and the nano-scale roughness on the microstructure side-wall prevents liquid intrusion, preventing the wetting-transition^64^. In conclusion, surface chemistry and roughness on the top of the microstructure determines the droplet evaporation mode and the micropillar side-wall’s surface chemistry, roughness, and cross-sectional shape determines its resistance to the wetting-transition.

The droplet wetting is strongly dependent on the nature of the surface (normal or sticky). During deposition, the droplet is able to retract multiple times on a normal superhydrophobic surface before attaining a stable position (Supplementary Movie 16) whereas the droplet adheres to the sticky superhydrophobic surface once it becomes into contact with it (Supplementary Movie 17). On sticky superhydrophobic surfaces the apparent contact angles are strongly affected by the method of droplet deposition. Also, it was previously reported that air-trapping is not possible in an open configuration (like the patterned microstructures)^38,70^. The higher apparent contact angles observed with very low area-fractions are due to the suspension (or overhanging) of the large droplets^71^ (Supplementary Movie 18, Fig. S-10) and not because of the trapped air-pockets.

Using a novel concept of critical FPL value, we explain the wetting-transition mechanism by balancing the Laplace pressure force and the capillary forces using a local force balance model. With the critical FPL value, we provide a method for the comparison of various superhydrophobic surfaces. A new model is developed relating the droplet physics to the solid-mechanics using a stiffness parameter “k_L_” which estimates the liquid’s response (deflection) to applied pressures. The stiffness parameter, together with the critical FPL value determine the wetting stability of the superhydrophobic surfaces. With a simple derivation, the relation between the contact line-fraction and area-fraction is explained and a conclusion is made that the area-fraction is a good estimate of the initial contact line-fraction. The calculated area-fractions on the line-shaped microstructures deviated from the theoretical area-fractions by a considerable margin, highlighting the drawbacks of Cassie-Baxter equation. The apparent contact angles, receding contact angles, and the droplet evaporation modes are explained successfully using contact line-fraction, while avoiding the capillary bridging effect. These findings provide one of the perspectives for understanding the wetting nature of surfaces, enabling design and engineering of pressure-stable superhydrophobic surfaces.

## Methods

### Fabrication of superhydrophobic surfaces

The patterned CNTs were initially grown by thermal CVD in a quartz tube furnace (Thermo-Fisher Minimite, 22 mm inner diameter). The catalyst for CNT growth is patterned on a (100) silicon wafer with 300 nm of thermally grown silicon dioxide by lift-off processing using photolithography, followed by ultrasonic agitation in acetone. The catalyst layer, 10 nm of Al_2_O_3_ and 1 nm of Fe, were sequentially deposited by electron beam physical vapor deposition. The wafer with the deposited catalyst is diced into ~2 × 2 cm pieces and placed in the quartz tube furnace for CNT growth. The growth recipe starts with flowing 100/400 s.c.c.m. of He/H_2_ while heating the furnace up to 775 °C over 10 min (ramping step); then, held at 775 °C for 10 min with the same gas flow rates (annealing step). Then the gas flow was changed to 100/400/100 s.c.c.m. of C_2_H_4_/He/H_2_ respectively at 775 °C for CNT growth for the selected duration. The typical growth rate is ~100 μm/min. After the growth, the furnace is cooled down to <100 °C at the same gas flow and finally purged with 1000 s.c.c.m. of He for 5 min.

### iCVD polymerization

A custom-built cylindrical reactor is used to perform iCVD polymerization (diameter 24.6 cm and height 3.8 cm). An array of 14 parallel Chromalloy filaments (Goodfellow) held around 2 cm above the reactor stage, where the growth substrates are kept, and is used to heat the initiator (*tert*-butyl peroxide, TBPO, 98% Aldrich) during polymerization. A quartz top (2.5 cm thick) covers the reactor allowing real-time thickness monitoring via reflecting a 633 nm He–Ne laser source (JDS Uniphase) off the substrate/polymer and recording the interference signal intensity as a function of time. A mechanical Fomblin pump (Leybold, Trivac) is used to lower the pressure inside the reactor and an MKS capacitive gauge is used to monitor the pressure. *1 H, 1 H, 2 H, 2H*-perfluorodecyl acrylate (PFDA, 97%) and the TBPO (98%) are used as received from Sigma Aldrich without any processing. A mass flow controller (1479 MFC, MKS Instruments) is used to adjust and deliver the TBPO at a constant flow rate of 1 s.c.c.m. A DC power supply (Sorensen) is utilized to heat the filament to the desired temperature (*T*_f_ = 250 °C). At this filament temperature, the labile peroxide bond of the TBPO breaks and creates –TBO radicals. The PFDA monomer is vaporized inside a glass jar through heating of the jar to a temperature of 80 °C, and then introduced to the reactor in the vapor phase through a needle valve at a constant flow rate of 0.2 s.c.c.m. The temperature of the growth substrate was maintained at *T*_s_ = 30 °C during polymerization using a recirculating chiller/heater (NESLAB RTE-7). K-type thermocouples (Omega Engineering) are used for measuring all of the temperatures. A throttle valve (MKS Instruments) is used to maintain a pressure of 60 mTorr during the polymerization. A silicon wafer is used as a control substrate during the PFDA polymerization; the thickness of the pPFDA deposited within approximately 30 minutes on a silicon substrate is approximately 30 nm as measured using ellipsometry.

### Droplet imaging

Water (DI water, Sigma-Aldrich) droplets of 4 µL are deposited on the substrates using with an automated dispenser (Model P/N 100-22, ramé-hart). The images of the droplet are captured using Nikon (D5500) DSLR camera with micro zoom lens (Navitar 2x F-mount). The contact angles are measured using ImageJ. Each contact angle value is averaged from measurements on ten discrete droplets distributed across the sample. Images are captured rapidly (less than 10 s) after droplet deposition to minimize impact of air exposure on the liquid droplets. For droplet evaporation studies, images are captured at regular intervals (10 s) and used for measuring the receding contact angles, contact line-fractions and for making videos. Ethylene glycol (Simga-Aldrich) droplet (6 μL) is used for the ESEM studies for observing the capillary bridging. The SEM and ESEM images are captured using a scanning electron microscope (Quanta 3D FEG).

### Slow-motion videos

The slow-motion videos of droplet deposition and impact tests are captured using a high-speed camera (OLYMPUS, i-SPEED TR) capable of capturing videos at 10,000 fps.

### Contact line-fraction

The contact line-fractions are calculated using a computer software (AutoCAD) by replicating the pattern dimensions and the droplet positions. The contact line-fractions and the theoretical area-fractions are calculated using the microscale roughness alone.

### Von Mises stress simulations

The solid modelling and the von Mises stress simulations are performed using COMSOL Multiphysics software.

## Supplementary information

Supplementary Information

Video M1

Video M2

Video M3

Video M4

Video M5

Video M6

Video M7

Video M8

Video M9

Video M10

Video M11

Video M12

Video M13

Video M14

Video M15

Video M16

Video M17

Video M18
