# Supplementary material for: Explaining Evaporation-Triggered Wetting Transition Using Local Force Balance Model and Contact Line-Fraction
Source: Sci Rep. 2019 Jan 23;9:405. doi: 10.1038/s41598-018-37093-6 (PMC6344486; doi:10.1038/s41598-018-37093-6)
Supplement: Supplementary file 1 — Supplementary Information [file 41598_2018_37093_MOESM1_ESM.docx]

**Supporting Information for**

**Explaining Evaporation-Triggered Wetting Transition Using Local Force Balance Model and Contact Line-Fraction**

Rama Kishore Annavarapu,^1^ Sanha Kim,^2^ Minghui Wang,^3^ A. John Hart,^2^ & Hossein Sojoudi^1*^

^1^Department of Mechanical, Industrial, and Manufacturing Engineering (MIME), The University of Toledo, 4006 Nitschke Hall, Toledo, OH, 43606, United States. ^2^Department of Mechanical Engineering, Massachusetts Institute of Technology (MIT), 77 Massachusetts Avenue, Cambridge, MA 02139, United States. ^3^Department of Chemical Engineering, Massachusetts Institute of Technology (MIT), 77 Massachusetts Avenue, Cambridge, MA 02139, United States.

**Author information**

Corresponding Author: ^*^E-mail: hossein.sojoudi@utoledo.edu;

**List of Contents**

**VIDEOS:**

**Videos M1-M4.** Show the evaporation of a water droplet (4 μL) on the pPFDA-coated CNT micropillars with area-fraction (f) = 0.03 with varying heights (H) of 40 μm, 50 μm, 60 μm, and 70 μm respectively (Playback at 100x).

**Videos M5-M8.** Show the evaporation of a water droplet (4 μL) on the pPFDA-coated CNT micropillars with varying area-fractions (f) of 0.12, 0.19, 0.28, and 0.38 respectively (Playback at 100x).

**Videos M9-M10.** Show the evaporation of a water droplet (4 μL) on the pPFDA-coated line-shaped CNT microstructures with area-fraction (f) = 0.09 with varying heights (H) of 30 μm, and 55 μm respectively (Playback at 100x).

**Video M11.** Show the evaporation of a water droplet (4 μL) on the pPFDA-coated line-shaped CNT microstructures with area-fraction (f) = 0.06 and height (H) of 55 μm (Playback at 100x).

**Videos M12-M13.** Show the bouncing of a water droplet (6 μL) on the pPFDA-coated line-shaped (captured at 500fps) and cylindrical CNT microstructures (captured at 750 fps) respectively.

**Videos M14-M15.** Video of the sequential environmental SEM images captured during the initial and later stages of evaporation of an ethyleneglycol droplet (initial droplet volume of 6 μL) on the pPFDA-coated CNT micropillars showing the effect of capillary bridging on the droplet retraction from the top of the micropillars.

**Videos M16-M17.** Show the deposition of the water droplet (4 μL) on normal and sticky pPFDA-coated CNT micropillars respectively (captured at 750 fps).

**Videos M18.** Show the overhanging portion of suspended water droplet (4 μL) on pPFDA-coated line-shaped CNT microstructures (captured at 750 fps).

**FIGURES:**

**Figure S-1.** The effect of micropillar height on the wettability of the pPFDA-coated CNT micropillars.

**Figure S-2.** Schematic showing the force zones across the droplet base.

**Figure S-3.** SEM images of the pPFDA coated CNT micropillars showing the re-entrant shape at the top.

**Figure S-4.** Schematic showing the shape angle of the micropillars and its effect on the critical FPL value.

**Figure S-5.** Graphic showing the effect of micropillar cross-sectional shape on the force distribution (or load sharing) and on the Cassie-Baxter stability.

**Figure S-6.** Plots showing the relation between the deflection (δ) and the Laplace pressure (P_L_) on cylindrical and line-shaped CNT microstructures.

**Figure S-7.** Images of the as-deposited water droplets (4 μL) on the pPFDA-coated CNT micropillars with varying area-fractions (f) and the schematics showing the relation between the area-fraction and the line-fraction.

**Figure S-8.** Schematic showing the differences in the theoretical area-fraction and the calculated area-fraction values on cylindrical and line-shaped microstructures, and the line-fraction.

**Figure S-9.** The relation between the contact-line fraction and the droplet evaporation modes.

**Figure S-10.** The effect of the overhanging portion of suspended droplet on the apparent contact angle.

**TABLES:**

**Table S-1.** Similarities between the area-fraction (f), contact line-fraction (L_1_) and the effective pinned fraction (Ф).

**Table S-2.** The effect of the contact line-fraction on the apparent (θ^app^) and the receding (θ^rec^) contact angles.


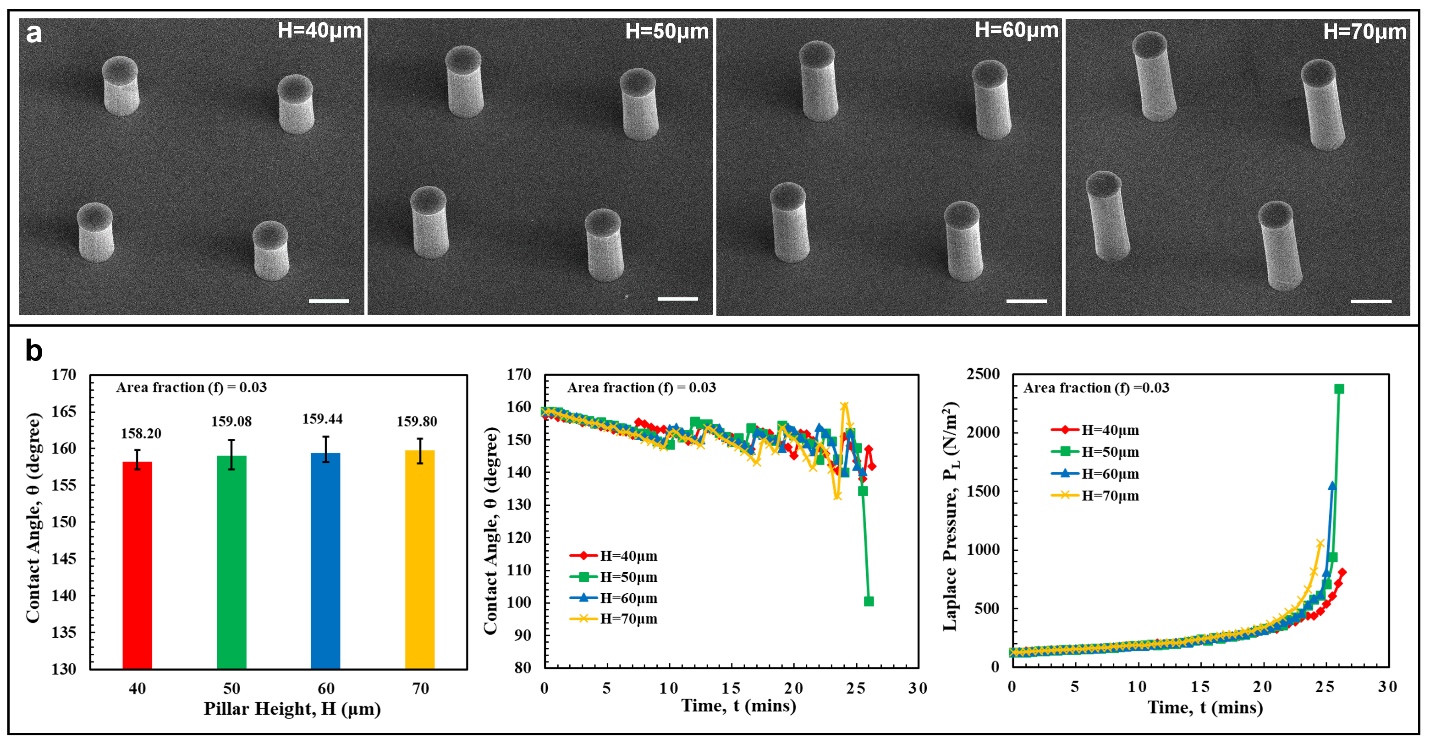


**Figure S-1. The effect of the micropillar height (H) on the wettability of the nanoporous CNT microstructures.** (**a**) SEM images of the pPFDA-coated VA-CNTs micropillars (radius, r =10 μm, center-to-center spacing, S =100 μm) with heights, H of 40 μm, 50 μm, 60 μm and 70 μm (left to right). (**b**) Plots showing the variation in the apparent water contact angle, θ with water, 4μL${(\gamma}_{\mathrm{Water}}=72.5 mN/m)$ (left panel) and the variation in the contact angle, θ (middle panel) and the Laplace pressure, P_L_ (right panel) during water droplet (4μL) evaporation. A stable state of wettability is observed with micropillar heights, H of 50 μm, 60 μm and 70 μm. The scale bar in (a) is 50 μm. Area fraction, f = 0.03 (using f=$\frac{\pi r^{2}}{S^{2}}$).


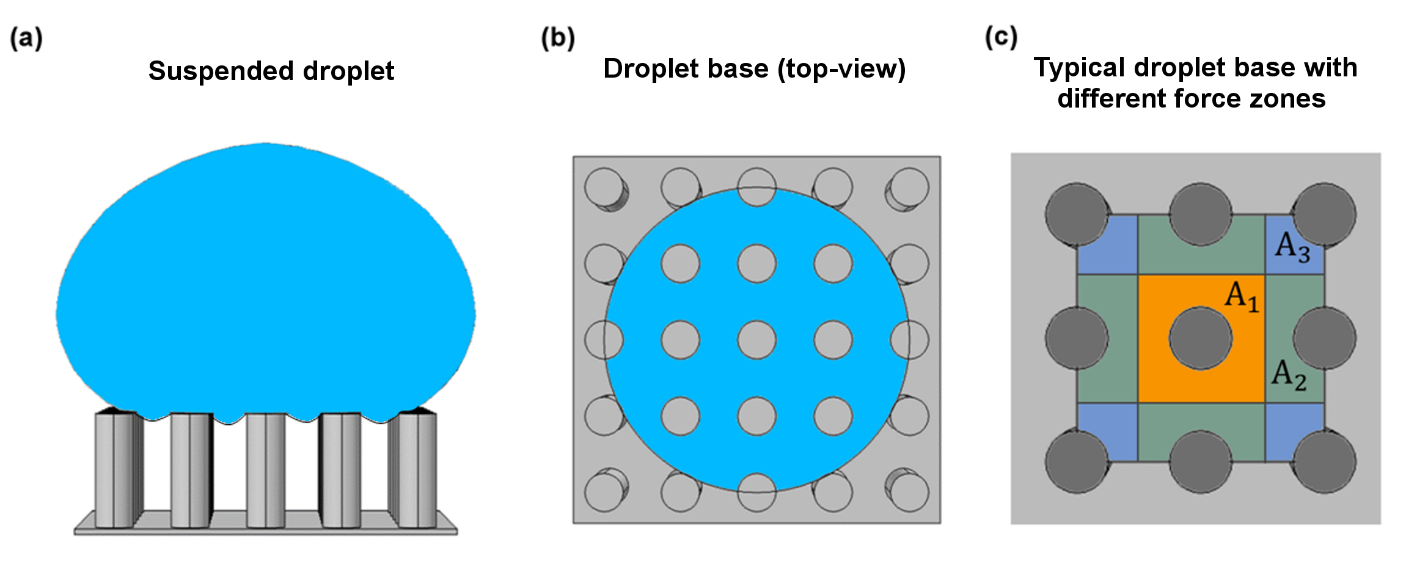


**Figure S-2. Force zones across the droplet base.** Schematic showing a suspended Cassie-Baxter state liquid droplet on the top of the micropillars (**a**) front view, and (**b**) top view (showing only the droplet base). (**c**) Schematic of a typical droplet base showing the different force zones across the droplet base. The downward force due to the Laplace pressure (P_L_) acting along the circumference of the micropillar is proportional to area covered by that micropillar. The micropillar in zone A_1_ (unit-cell) experiences higher downward force than the peripheral micropillars in the zones A_2_ and A_3_. The whole droplet base can be divided into different force zones accordingly.


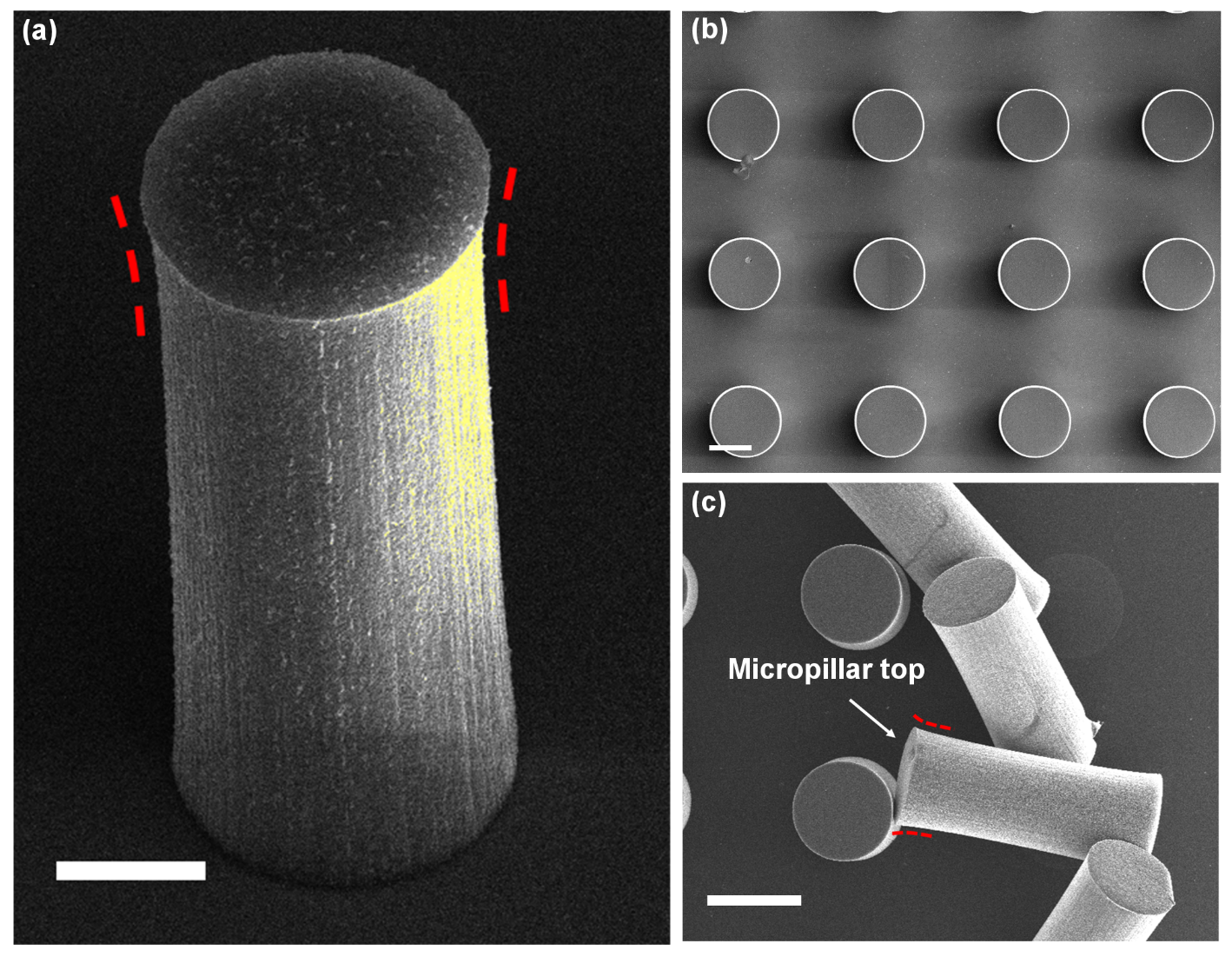


**Figure S-3. Re-entrant shape of the pPFDA-coated CNT micropillar.** (**a**) SEM imaged of the pPFDA-coated CNT micropillar showing the re-entrant shape (red dotted-line) at the top. The scale bar is 10 μm. (**b**) SEM image showing the top-view of the patterned micropillars. The white circles around the micropillars are due to the difference between the diameters at the top and bottom of the micropillar, resembling the re-entrant shape. (**c**) SEM image showing the re-entrant shape (red dotted-line) on distorted micropillars. The scale bar in (**b**) and (**c**) is 50 μm. The re-entrant shape increases the stability to liquid-pressure and hence increases the critical FPL value.


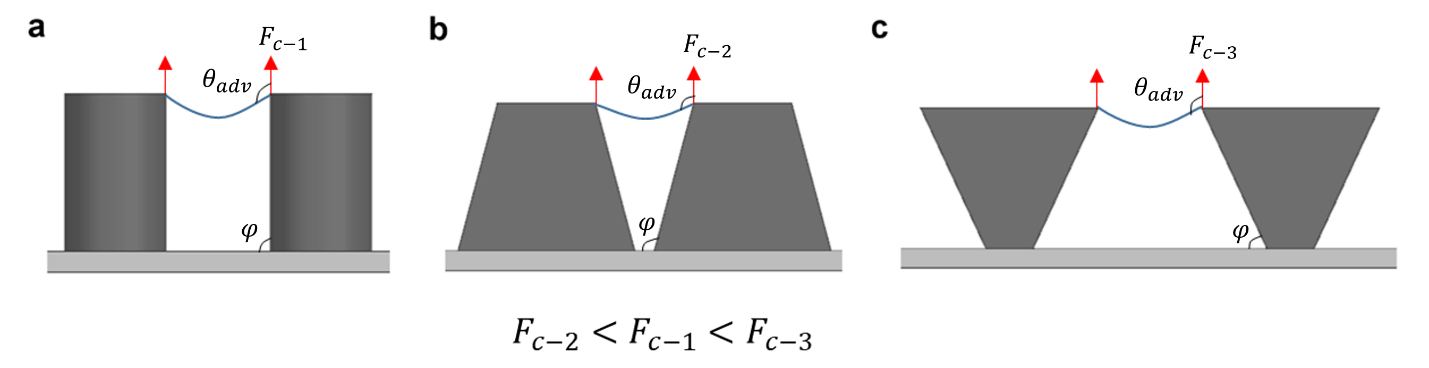


**Fig S-4. The effect of the micropillar shape on the critical FPL value.** Schematic showing the micropillars with different shape angle ($\varphi$), (**a**) $\varphi$ = 90°, (**b**) $\varphi$ > 90° and (**c**) $\varphi$ < 90°. The vertical capillary force given by $\gamma_{L}Cos(90+\varphi-\theta^{\mathrm{adv}})$ decreases with increasing shape-angle ($\varphi$) of the micropillar. The re-entrant shape increases the Cassie-Baxter droplet stability by resisting the wetting transition.


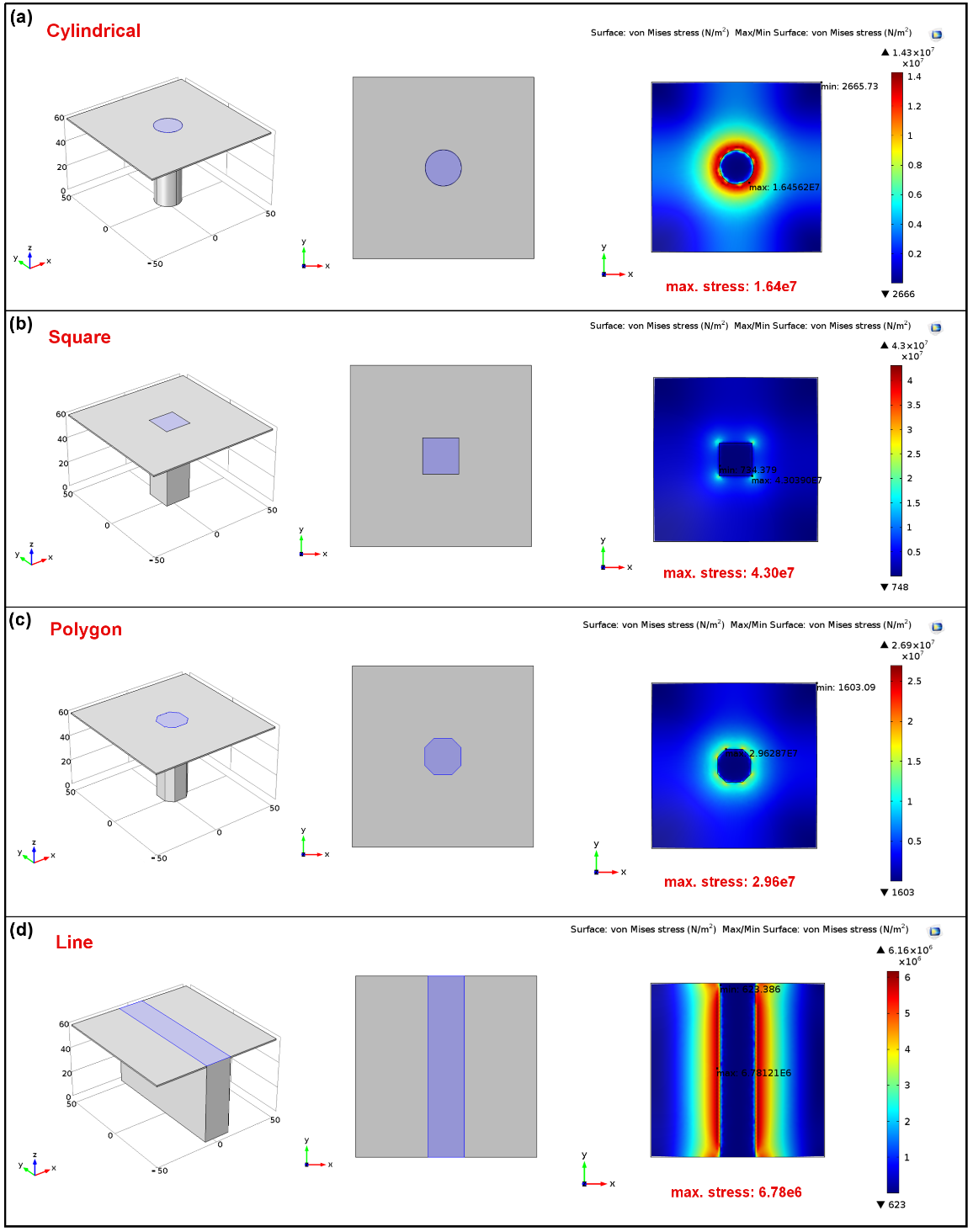


**Fig S-5. The effect of the microstructure cross-sectional shape on the force distribution (or load sharing) and on the Cassie-Baxter state stability.** Graphic showing the stress distributions for different micropillar cross-sectional shapes (with same width (or diameter) = 20 μm and height H = 60 μm), (**a**) Cylindrical, (**b**) Square, (**c**) Polygon, and (**d**) Line-shaped. A unit-cell size of 100 μm x 100 μm is considered and pressure of 1500 N/m^2^ is applied downward (negative Z direction) to resemble the Laplace pressure (P_L_). It is observed that the maximum von Mises stress values are smaller for Line-shaped and the Cylindrical microstructures due to the more uniform force distribution (or load sharing) along the circumference. Moreover, the maximum von Mises stress on the Line-shaped microstructure is smaller than that on the Cylindrical microstructure, meaning that they have higher pressure stability which is in accordance with the droplet evaporation experiments.


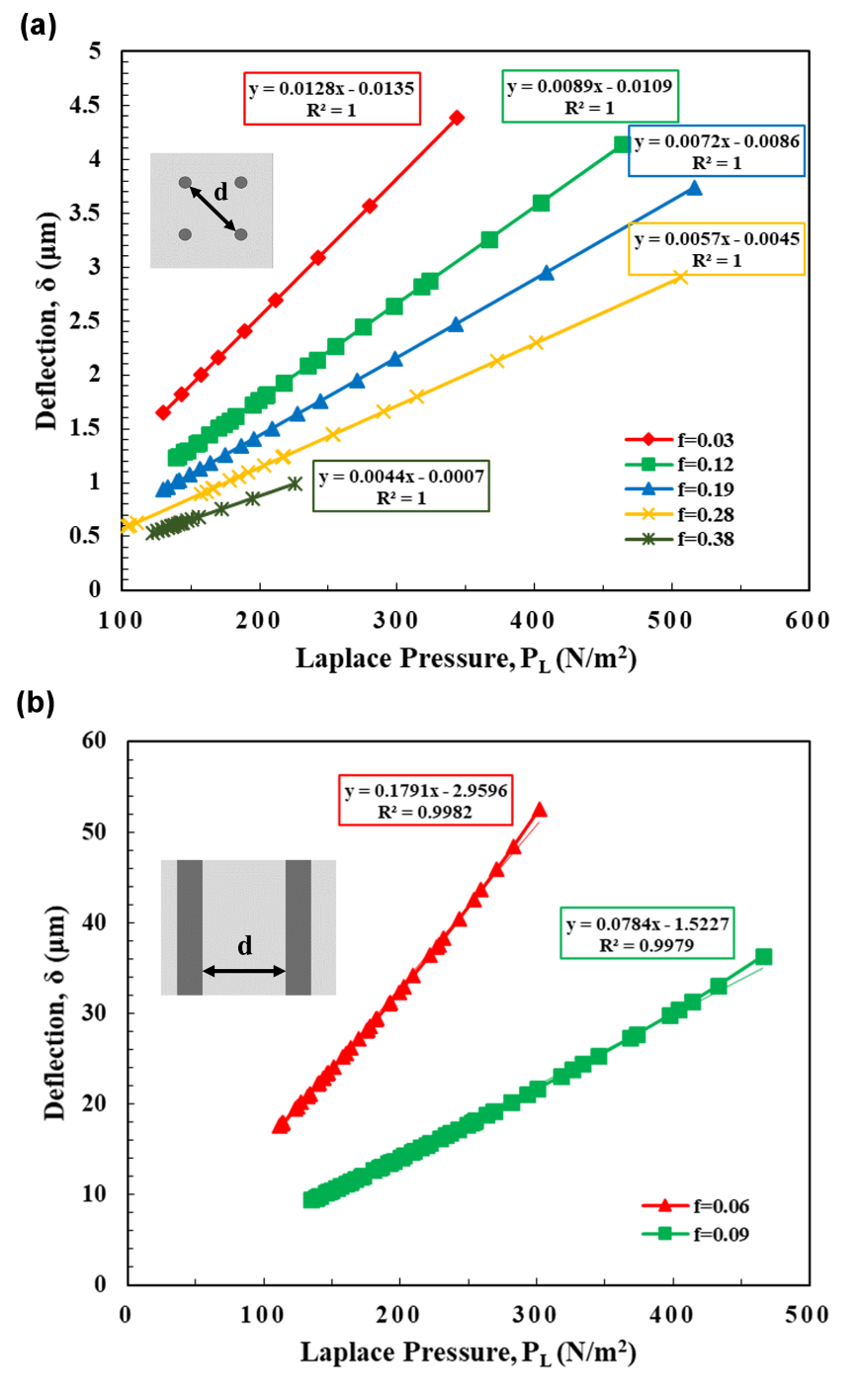


**Figure S-6.** **The relation between the deflection (δ) and the Laplace pressure (P_L_).** Plots showing the variation of the deflection (δ) value with the Laplace pressure (P_L_) for different area-fractions (f) during the evaporation of a water droplet (4μL) on cylindrical (**a**) and line-shaped (**b**) microstructures. At lower area-fractions the deflection (δ) values are higher and a linear relation is observed between the deflection (δ) and the Laplace pressure (P_L_).


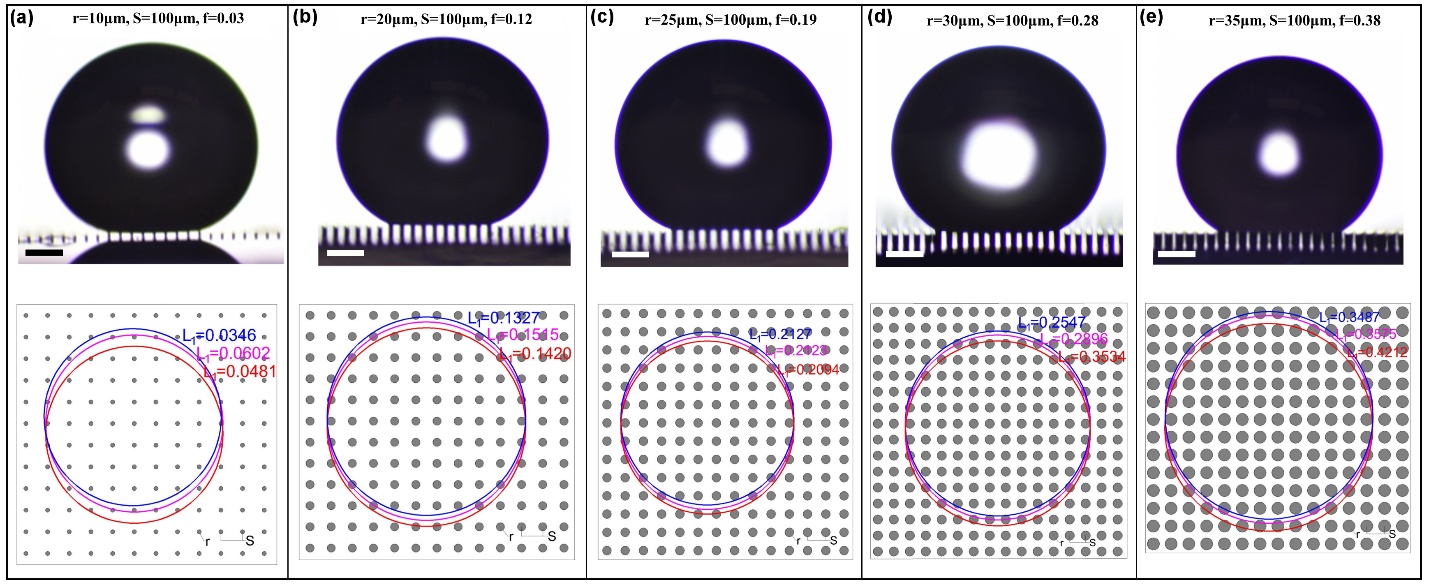


**Figure S-7. The relation between contact line-fraction (L_1_) and area-fraction (f).** (**a**), (**b**), (**c**), (**d**) and (**e**) Showing the images of the as-deposited water droplet (4μL) (**top**) and the corresponding contact line-fractions (**bottom**). The contact line-fractions are calculated for the possible droplet states (shown in blue, magenta and red color). The line-fractions are in close approximation with the theoretical area-fraction increasing their chances for use in the contact angle equation.


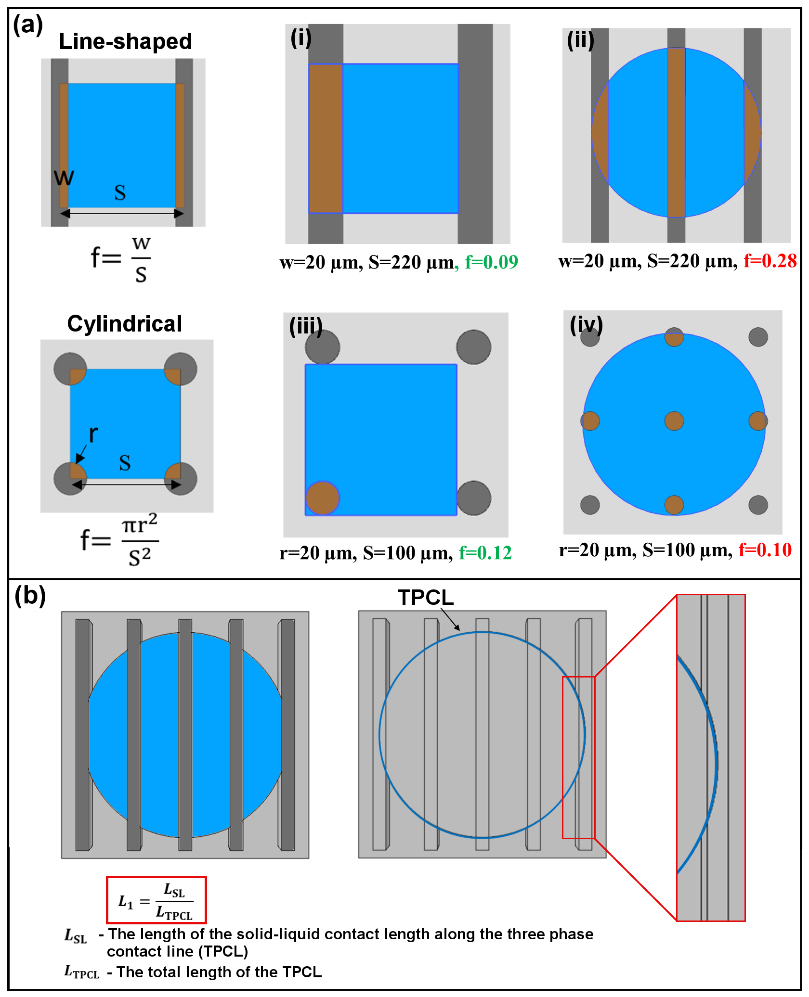


**Figure S-8.** (**a**) Schematic showing the unit-cell droplet configuration for the line-shaped and the cylindrical microstructures and the formulas for calculating the area-fraction (f) based on the solid-liquid contact area (brown). (**i**) and (**iii**) show a typical example where the area-fraction (f) is calculated using the present models. (**ii**) and (**iv**) show the values of the area-fraction (f) calculated based on the actual solid-liquid contact area. A large difference is observed in the area-fraction (f) between the calculated and the actual values for the line-shaped microstructures, indicating short-comings of available models. (**b**) Schematic showing the droplet base (left panel), the three-phase contact line, TPCL (middle panel) and the zoomed section of the TPCL (right panel). At the edge of the TPCL, The solid-liquid contact length is higher (approximately 2-3 times) than the microstructure width (w), leading to an increase in the area-fraction (f). Alternatively, pinned contact line-fraction (L_1_) can be used instead of the area-fraction (f).


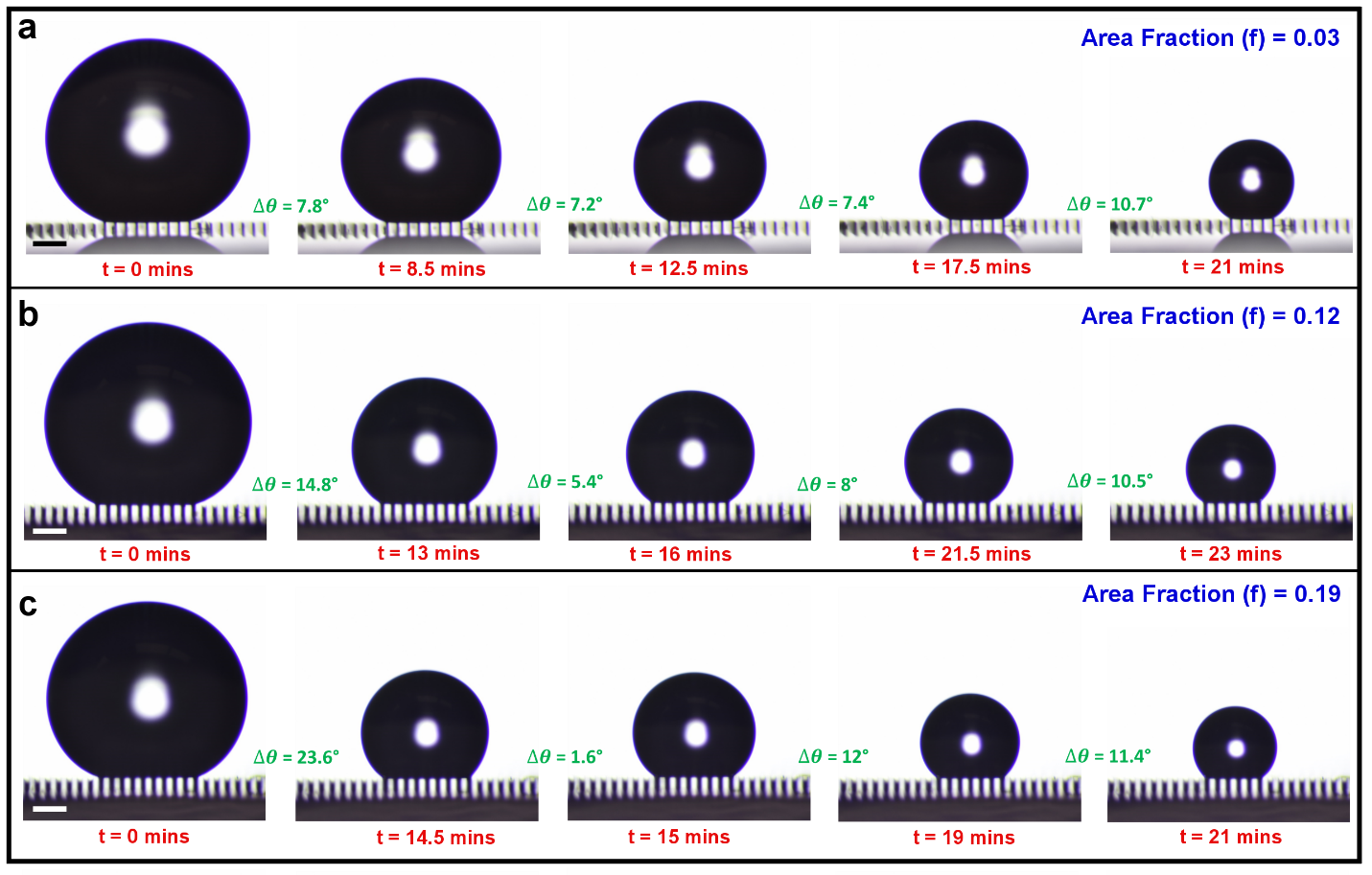


**Figure S-9. Relation between contact-line fraction and the droplet evaporation modes.** Images showing the evaporation of water droplets (4μL) on samples with varying area fractions. (**a**) f = 0.03, (**b**) f = 0.12, and (**c**) f = 0.19. ∆θ represents the change in the contact angle (from its apparent contact angle to the receding contact angle) required for a droplet state to retract from the micropillar. The higher the ∆θ, the more the time it takes for retraction causing CCR evaporation mode. For smaller area-fraction (**a**) the droplet retracted early (t = 8.5 mins) than (**b**) t = 13 mins and (**c**) t = 14.5 mins due to smaller ∆θ, exhibiting CCA evaporation mode. The same is applicable for the remaining part of the evaporation. The stick-slip mechanism observed during droplet evaporation is a result of ∆θ required for TCPL depinning.


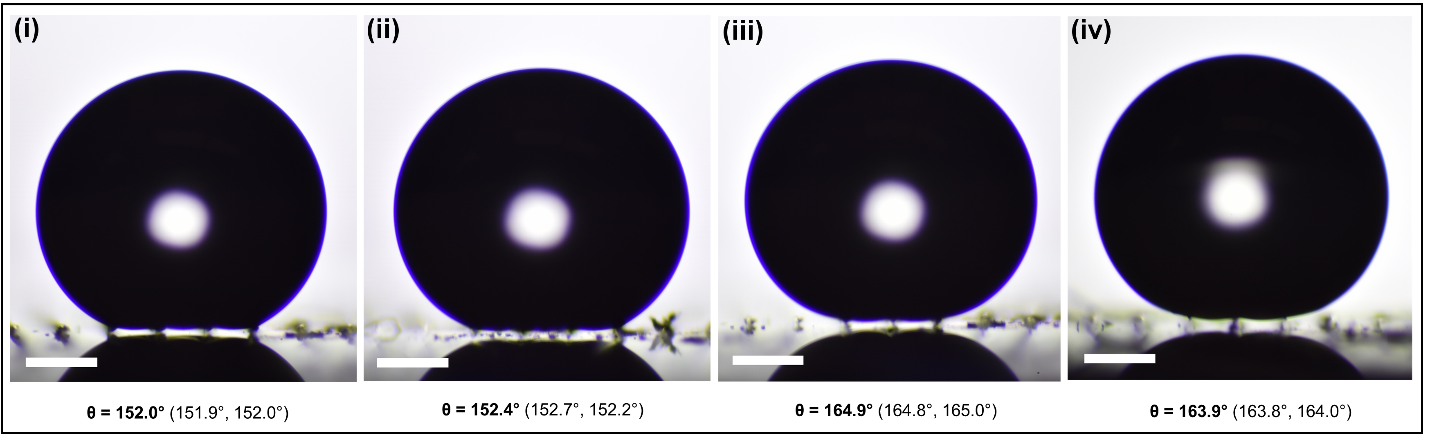


**Figure S-10. The overhanging portion of a suspended droplet.** Images of the as-deposited water droplet (4 μL) on line-shaped microstructures with width w =20 μm and center-to-center spacing S =320 μm (f=0.06). A suspended droplet state is observed with (**i**), (**ii)** four and (**iii**), (**iv**) three microstructures beneath the droplet with apparent contact angles (θ) of 152.2°±0.5° and 163.9°±1.0° respectively. The higher contact angle with three microstructures is due to the hanging of the extra volume of the droplet. For very low area-fractions the apparent contact angles are effected by the way of droplet deposition.

**Table S-1. Similarities between area-fraction (f), contact line-fraction** $\boldsymbol{(L}_{\mathbf{1}}\boldsymbol{)}$**and effective pinned fraction (**$\boldsymbol{\Phi}\boldsymbol{)}$**.** Contact line-fractions are calculated for three (assumed) droplet positions on the top of the micropillars (See Supplementary Fig. S-7). The calculated line-fractions (L_1_) are very close to the theoretical area-fractions (f). Different contact angles observed during droplet deposition are due to the differences in the contact line-fractions. However, the calculated effective pinned fraction (Φ) values are much higher than the theoretical area-fractions and contact line-fractions. Smaller deviations are observed at higher area-fractions.

**Cylindrical Micropillars** (center-to-center spacing (S) =100μm)

|  | **Area fraction (f**$\boldsymbol{)}$ | **Contact line-fraction (**$\boldsymbol{L}_{\boldsymbol{1}}\boldsymbol{)}$ | **Effective pinned fraction (**$\boldsymbol{Ф)}$ |
| --- | --- | --- | --- |
| Radius (r) =10μm | 0.03 | 0.0346  0.0481  0.0602 | 0.62 |
| Radius (r) =20μm | 0.12 | 0.1327  0.1420  0.1515 | 1.25 |
| Radius (r) =25μm | 0.19 | 0.2094  0.2123  0.2127 | 1.57 |
| Radius (r) =30μm | 0.28 | 0.2547  0.2896  0.3534 | 1.88 |
| Radius (r) =35μm | 0.38 | 0.3487  0.3575  0.4212 | 2.19 |

**Table S-2. The effect of contact line-fraction on the apparent and receding contact angles.** The apparent and receding contact angles are measured for the first two retractions using imageJ. Table shows the average contact angle along with the left and right side contact angle values of the droplet. The apparent contact angles and the receding contact angles decreased during droplet evaporation with an increase in the contact line-fractions revealing their dependency on the contact line-fraction (see Fig. 8). The variation in the contact angle measurements is ±0.5°.

Width w = 20 μm, Center-to-center spacing S = 220 μm, area-fraction f = 0.09

| **Experiment runs** | **Length Fraction (**$\boldsymbol{L}_{\mathbf{1}}\mathbf{)}$ | **Apparent contact angle (**$\boldsymbol{\theta}^{\mathbf{app}}\mathbf{)}$ | **Receding contact angle (**$\boldsymbol{\theta}^{\mathbf{rec}}\mathbf{)}$ |
| --- | --- | --- | --- |
| 1^st^ run | 0.2371 | **156.1°** (156.0°, 156.2°) | **142.4°** (142.7°, 142.1°) |
|  | 0.2612 | **152.9°** (152.4°, 153.4°) | **139.0°** (139.4°, 138.6°) |
| 2^nd^ run | 0.2371 | **156.4°** (156.3°, 156.4°) | **141.2°** (141.7°, 140.7°) |
|  | 0.2612 | **153.6°** (153.1°, 154.0°) | **138.2°** (138.6°, 137.9°) |
